# Strategies to Improve Multi-enzyme Compatibility and Coordination in One-Pot SHERLOCK

**DOI:** 10.1021/acs.analchem.2c05032

**Published:** 2023-06-30

**Authors:** Hongzhao Li, Dominic M. S. Kielich, Guodong Liu, Greg Smith, Alexander Bello, James E. Strong, Bradley S. Pickering

**Affiliations:** † National Centre for Foreign Animal Disease, Canadian Food Inspection Agency, Winnipeg R3E 3M4, Manitoba, Canada; ‡ Department of Medical Microbiology and Infectious Diseases, College of Medicine, Faculty of Health Sciences, University of Manitoba, Winnipeg R3E 0J9, Manitoba, Canada; § National Microbiology Laboratory, Public Health Agency of Canada, Winnipeg R3E 3M4, Manitoba, Canada; ∥ Department of Pediatrics & Child Health, College of Medicine, Faculty of Health Sciences, University of Manitoba, Winnipeg R3A 1S1, Manitoba, Canada; ⊥ College of Veterinary Medicine, Department of Veterinary Microbiology and Preventive Medicine, Iowa State University, Ames, Iowa 50011, United States

## Abstract

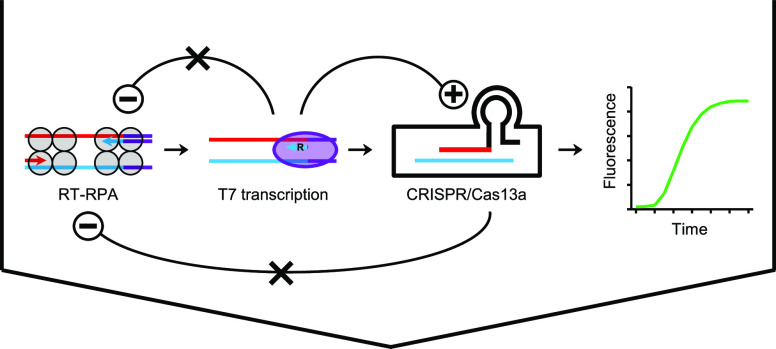

While molecular diagnostics generally require heating elements that supply high temperatures such as 95 °C in polymerase chain reaction and 60–69 °C in loop-mediated isothermal amplification, the recently developed CRISPR-based SHERLOCK (specific high-sensitivity enzymatic reporter unlocking) platform can operate at 37 °C or a similar ambient temperature. This unique advantage may be translated into highly energy-efficient or equipment-free molecular diagnostic systems with unrestricted deployability. SHERLOCK is characterized by ultra-high sensitivity when performed in a traditional two-step format. For RNA sensing, the first step combines reverse transcription with recombinase polymerase amplification, while the second step consists of T7 transcription and CRISPR-Cas13a detection. The sensitivity drops dramatically, however, when all these components are combined into a single reaction mixture, and it largely remains an unmet need in the field to establish a high-performance one-pot SHERLOCK assay. An underlying challenge, conceivably, is the extremely complex nature of a one-pot formulation, crowding a large number of reaction types using at least eight enzymes/proteins. Although previous work has made substantial improvements by serving individual enzymes/reactions with accommodating conditions, we reason that the interactions among different enzymatic reactions could be another layer of complicating factors. In this study, we seek optimization strategies by which inter-enzymatic interference may be eliminated or reduced and cooperation created or enhanced. Several such strategies are identified for SARS-CoV-2 detection, each leading to a significantly improved reaction profile with faster and stronger signal amplification. Designed based on common molecular biology principles, these strategies are expected to be customizable and generalizable with various buffer conditions or pathogen types, thus holding broad applicability for integration into future development of one-pot diagnostics in the form of a highly coordinated multi-enzyme reaction system.

## Introduction

Quantitative polymerase chain reaction (qPCR)-based methods are the current gold standard for the diagnosis of early-stage infections but have limitations (detailed in Introduction S1). As potential supplements or alternatives, a number of isothermal molecular detection platforms, particularly those based on CRISPR (clustered regularly interspaced short palindromic repeats), are opening a new frontier in point-of-need (PON) testing.^1−4^ In a CRISPR diagnostic reaction, a CRISPR RNA (crRNA or guide RNA) and a CRISPR-associated (Cas) protein effector (Cas13a, Cas12a, or Cas12b) form a surveillance complex, where a spacer (guide) sequence of the crRNA specifically complements the target nucleic acid sequence and bridges the activation of the Cas protein. An activated Cas protein not only degrades its specific targets but also exhibits a collateral and indiscriminate RNase (Cas13a) or DNase (Cas12a and Cas12b) activity that trans-cleaves the bystander RNA or DNA reporter molecules, leading to enormous amplification of fluorescent or colorimetric signals. Current CRISPR diagnostics typically combine isothermal nucleic acid amplification often by recombinase polymerase amplification (RPA) or loop-mediated isothermal amplification (LAMP) with CRISPR-Cas nuclease-mediated signal amplification and detection.^4−6^ Such combinations lead to greatly enhanced diagnostic specificity and sensitivity, which are similar to those achievable by qPCR.

Depending on the type of Cas enzymes involved, these diagnostic systems are frequently termed SHERLOCK (specific high-sensitivity enzymatic reporter unlocking) if using Cas13 to recognize an RNA CRISPR substrate or DETECTR (DNA endonuclease-targeted CRISPR trans reporter) if using Cas12 to recognize a DNA CRISPR substrate.^7,8^ The traditional definition of SHERLOCK is being extended by some researchers to any assay combining isothermal amplification and CRISPR detection, while the definition of DETECTR remains as an assay using Cas12a in combination with isothermal amplification.^9,10^ Here, in this study, we follow the original definition of SHERLOCK (as a major prototype system representing CRISPR diagnostics), specifically based on the formulation first published in 2017.^7^ Accordingly, a SHERLOCK assay integrates RPA, T7 transcription, and CRISPR/Cas13a, three major known molecular amplification techniques that can be performed at or around 37 °C.^9^ This temperature range awards SHERLOCK a particular advantage over other nucleic acid detection methods, which usually run at higher temperatures such as 95 °C in PCR and 60–69 °C in LAMP, thereby requiring (often sophisticated and expensive) equipment with heating capacity and electricity supply.^11,12^ Since SHERLOCK could simply be powered by body heat or in any environment with a similar temperature, it holds a unique potential for simple, power/equipment-free, highly field-deployable testing and represents a new generation of molecular diagnostic solutions to a broad range of PON scenarios. One example is an extremely fast-spreading outbreak such as the Omicron wave of the COVID-19 pandemic where the demand for quick and widespread testing overwhelmed the capacity of PCR-based diagnostics.^13^ Another scenario is an outbreak in resource-limited settings, where a lack of early diagnosis could result in failure to contain further spread. Indeed, outbreaks often emerge or have the potential to emerge from developing or remote areas.^14−19^ Moreover, pathogens known or expected to cause outbreaks can often emerge from a zoonotic source especially from wildlife in rural or remote locations.^14−21^ These include major high-impact pathogens such as Ebola virus, SARS-CoV-2 and its related family SARS-CoV (severe acute respiratory syndrome coronavirus) and MERS-CoV (Middle East respiratory syndrome coronavirus), and Zika virus.^14−19^ Proactive, on-site SHERLOCK testing in reservoir and intermediate animal hosts could be utilized to greatly facilitate surveillance and research activities contributing to public health preparedness for potential future outbreaks. In addition, SHERLOCK has other advantages (Introduction S1).

SHERLOCK is traditionally run as a two-step process. For RNA virus detection, step 1 is a combination of reverse transcription (RT) to convert RNA into DNA and RPA to amplify DNA (RT-RPA). Step 2 uses T7 transcription to convert and amplify DNA into a target RNA to be recognized by and subsequently activate CRISPR/Cas13a, which in turn cleaves reporter molecules to generate amplification signals.^9,22^ Ideally, a one-step SHERLOCK format would minimize reagent handling steps, reduce user errors and risk of contamination, and save time, thus making the assay more user-friendly, efficient, and deployable. However, simply combining all the ingredients from the two steps into one single mixture results in severe reduction in sensitivity by a factor of several orders of magnitude.^23^ Optimization of one-pot SHERLOCK has remained a challenging task, with a limited number of assays showing substantially improved sensitivity near the range of 100 template copies/μL (cp/μL), including the SHINE, SHINEv2, MEDICA, and S-PREP/SHERLOCK assays.^23−26^ Although not having reached the sensitivity of two-step SHERLOCK and qPCR, typically at the range of 1 cp/μL, this initial progress is encouraging and is anticipated to be extended if new, effective optimization strategies can be ascertained.

In simpler diagnostic systems such as (RT-)qPCR that involve one or two enzymes (Taq polymerase ± RT), the scope of optimizations can be all covered by those directed at the individual enzyme(s), while inter-enzyme interactions do not exist or are negligible for consideration. Similar strategies on the level of approaching individual enzymatic reactions have contributed to improvement of one-pot SHERLOCK in the SHINE assays.^23,24^ However, it is perceivable that a one-pot SHERLOCK formulation is an extremely complex and crowded molecular system that involves a large number of reaction components including multiple enzymes/proteins (at least eight). The RT reaction needs an RT enzyme and a potential accessory enzyme, RNase H;^23,27^ RPA depends on a recombinase, recombinase-loading factor, single strand-binding protein, polymerase, and creatine kinase;^28^ T7 transcription and CRISPR reactions require a T7 polymerase and a Cas13a protein,^9^ respectively.

In light of the various complexities brought about by the numerous enzymatic reactions occurring simultaneously in a one-pot SHERLOCK assay, optimizations toward addressing such complexities are anticipated to enhance assay performance. Therefore, this study was delivered to test optimization strategies by which inter-enzymatic interference might be eliminated or reduced and cooperation of reactions could be created or enhanced. In this context, using SARS-CoV-2 as a target model pathogen, we identified several optimization strategies (overview in Introduction S1).

## Materials and Methods

In addition to the brief summary below, detailed information can be found in Materials and Methods S1.

### Optimization of One-Pot SHERLOCK

As a starting point of optimization, we assembled a base one-pot formulation by combining the reaction components typically found in traditional two-step SHERLOCK assays. The optimization was conducted iteratively through multiple rounds, with a single component added, deleted, or titrated in each round.^23^ The dilution of the base formulation with extra volume of water was the first optimization step, and all the other optimizations were carried out after this step. For all optimization experiments, the tested reagent is described in related figures or supplementary figures. The modification that generated the most optimal reaction kinetics (the fastest and strongest signal amplification) was incorporated into an updated protocol at the end of each optimization round. These steps eventually led to an optimized formulation as detailed below.

### Optimized Formulation of One-Pot SHERLOCK

A master mix (78 μL) was assembled on ice, consisting of the following components: one TwistAmp pellet, 29.5 μL TwistAmp rehydration buffer, 6.105 μL H_2_O, 24.445 μL protease buffer, 1.8 μL RPA primer mix (10 μM T7 promoter-tagged forward primer, 10 μM non-T7 forward primer, and 20 μM reverse primer), 1.25 μL RNase inhibitor (40 U/μL), 0.8 μL M-MuLV reverse transcriptase (200 U/μL), 1.6 μL RNase H (5 U/μL), 2.5 μL TwistAmp magnesium acetate (280 mM), 2.25 μL MgCl_2_ (200 mM), 2 μL rNTP mix (25 mM each), 1.25 μL T7 RNA polymerase (50 U/μL), 1.25 μL crRNA (100 ng/μL) or crDNA (0.05 μM), 2 μL U5 reporter (5 μM), and 1.25 μL LwaCas13a protein (126.6 ng/μL in LwaCas13a storage buffer). The 78 μL master mix was divided into 2 × 39 μL, each then receiving 1 μL SARS-CoV-2 RNA or control sample to complete a 40 μL one-pot SHERLOCK reaction.

## Results and Discussion

### One-Pot SHERLOCK with CRISPR Targeting the Opposite Polarity of the SARS-CoV-2 Genome Is Functional and Specific

**Figure 1:**
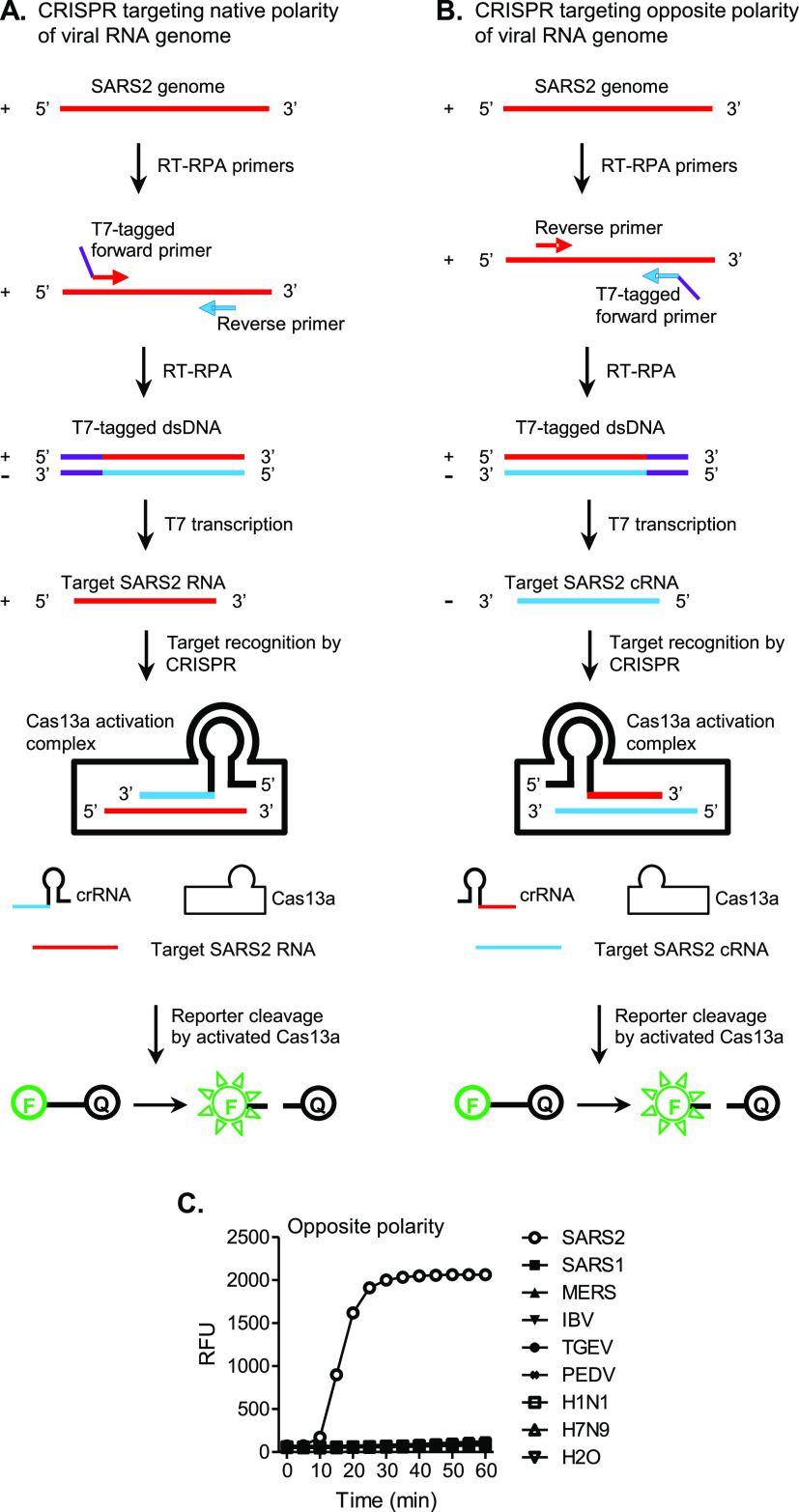
One-pot SHERLOCK with CRISPR targeting the opposite polarity of the SARS-CoV-2 (SARS2) genome is functional and specific. Schematics are not drawn to scale or necessarily reflect the exact molecular shapes. The polarity of a SARS2 sequence on an RNA or DNA strand is differentially indicated in red (native polarity = positive sense) versus blue (opposite polarity = negative sense). For simplicity, however, non-SARS2-like sequences are not color-differentiated for polarities, with both polarities shown in the same color. These sequences are represented in black, except that T7 promoter sequences are indicated in purple. In general, the polarity of an RNA or DNA strand, labeled as “+” (positive sense), or “–” (negative sense), was defined according to the polarity of the SARS2 sequence it contains. The native SARS2 genomic sequence (positive-sense RNA strand, indicated in red) is first converted and amplified by RT-RPA into a T7 promoter-tagged double-stranded DNA (T7-tagged dsDNA). The DNA is then T7-transcribed and amplified into a final RNA strand for CRISPR targeting. The resulting activation of Cas13a leads to the cleavage of an RNA reporter and the release of fluorescent signals. In the RNA reporter, “F” and “Q” mean fluorophore and quencher, respectively. The final RNA strand to be targeted by CRISPR can have a native SARS2 genome polarity (+/red, as in panel A) or an opposite polarity (−/blue, as in panel B), depending on the polarity of the SARS2 sequence in the RPA primer that the T7 promoter sequence is attached to. Corresponding to that, the crRNA will have a complementary spacer sequence (−/blue in A or +/red in B), in order to bind the target RNA. (A) Traditional SHERLOCK design, with CRISPR targeting the native polarity of the viral RNA genome. (B) New SHERLOCK design, with CRISPR targeting the opposite polarity of the viral RNA genome. (C) A set of RPA primers and crRNA based on the opposite polarity-targeting SHERLOCK design strategy (SHERLOCK set opORF1ab) was tested for functional and specific detection of SARS2 using a panel of coronaviruses and other respiratory viruses: SARS2, SARS1 (SARS-CoV), MERS (MERS-CoV), IBV (infectious bronchitis virus), TGEV (transmissible gastroenteritis virus), PEDV (porcine epidemic diarrhea virus), H1N1 (H1N1 influenza virus), and H7N9 (H7N9 influenza virus). Amplification plot shows relative fluorescent units (RFU) at indicated time points in the SHERLOCK reactions. Graph represents three independent experiments showing similar patterns.

In a SHERLOCK assay, a pair of primers, including one primer tagged with a T7 promoter (T7 Pro) sequence, are used in RT-RPA to amplify a T7 Pro-containing double-stranded DNA (dsDNA). The dsDNA is subsequently amplified by T7 transcription into a single-stranded RNA, which is the target RNA to be finally recognized by a crRNA. In theory, depending on which primer is tagged with a T7 Pro sequence, CRISPR can be directed either at a target RNA sequence with the native polarity found in the original viral RNA genome (Figure 1A) or at a complementary target RNA (cRNA) with the opposite polarity (Figure 1B). The native polarity-targeting CRISPR design, however, appears to be default in previously developed SHERLOCK assays.^23,29,30^ For detecting the natural SARS-CoV-2 genome, which has a positive-sense polarity, generally the T7 Pro sequence is attached to the RPA primer with a positive-sense SARS-CoV-2 sequence (Figure 1A). This leads to the production by T7 transcription of a final CRISPR target RNA of positive sense (the native SARS-CoV-2 polarity) to be recognized by a crRNA spacer of negative sense (the opposite SARS-CoV-2 polarity). Here, we tested the possibility to direct CRISPR at the opposite polarity of the viral RNA genome in one-pot SHERLOCK detection of SARS-CoV-2 (Figure 1B,C). The new assay adopted a previously published SHERLOCK set targeting ORF1ab except that the SARS-CoV-2-derived sequences in the RPA primers and crRNA were switched to their reverse-complementary counterparts, in order for CRISPR to be directed at the negative sense of the SARS-CoV-2 sequence (opposite to native polarity).^29^ These RPA primers and crRNA in combination were named SHERLOCK set opORF1ab, whereas its counterpart SHERLOCK set targeting the native polarity of the viral RNA genome was named the ORF1ab set. As the major and most frequently used set, opORF1ab is the default SHERLOCK set in the description of experiments unless any other SHERLOCK set is specified. The test of the opposite polarity-targeting strategy involved a negative target panel consisting of coronaviruses closely related to SARS-CoV-2 (SARS-CoV and MERS-CoV), other coronaviruses and influenza viruses representing other respiratory viruses, along with SARS-CoV-2. Signal amplification was effectively generated against SARS-CoV-2 but none of the targets from the negative panel (Figure 1C), demonstrating that one-pot SHERLOCK with CRISPR targeting the opposite polarity of the viral RNA genome is functional and specific and represents a viable new option for SHERLOCK design. Additionally, we discuss with supporting data the possibility that directing CRISPR at the opposite polarity of the viral genome may offer enhanced capacity of testing specificity and ease of crRNA sequence selection compared to targeting the native polarity (Results and Discussion S1 and Figures S1 and S2).

### Reactions Originating from One Step of the Two-Step SHERLOCK Assay May Inhibit Those Originating from the Other Step

**Figure 2:**
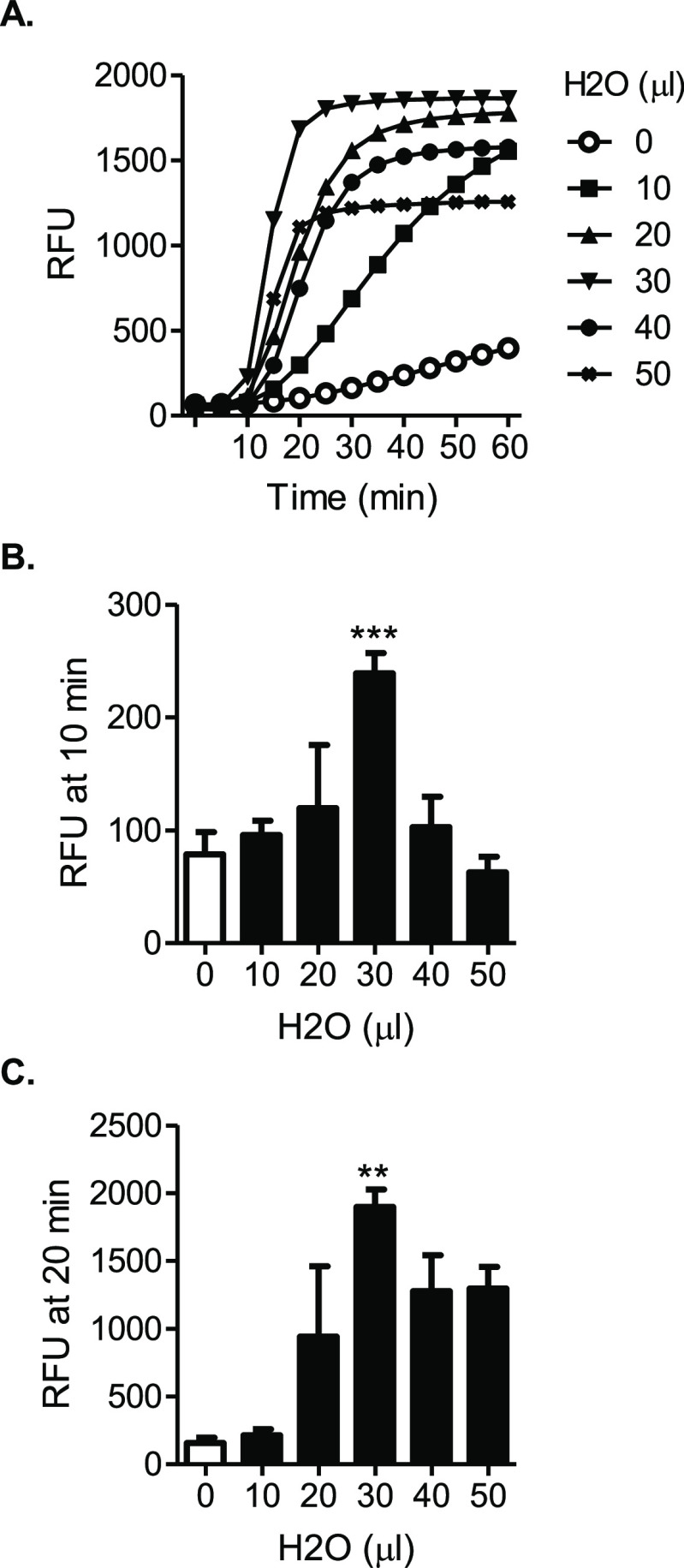
Addition of H_2_O enhances one-pot SHERLOCK. (A) H_2_O at each indicated volume was added to a 50 μL base one-pot SHERLOCK formulation (detailed in Materials and Methods S1). The effect of H_2_O addition was tested for SARS-CoV-2 detection. Amplification plot shows RFU at indicated time points in the SHERLOCK reaction and represents three independent experiments showing similar patterns. (B,C) Data from panel A were statistically analyzed, comparing the amplification signals between a condition with a positive volume of H_2_O added and the condition without H_2_O added (0 μL), at the time point of 10 min (B) or 20 min (C). Bar graphs indicate mean ± SEM from three independent experiments. Significant differences in RFUs were determined by paired *t*-test: ***p < 0.001 and **p < 0.01.

Although a one-pot SHERLOCK formulation is preferred over a two-step format, its development is challenging. A previous study showed that when all the components from the traditional two-step SHERLOCK assay were combined into a single reaction mixture, the assay sensitivity dropped by several orders of magnitude.^23^ This was similarly observed in our experiments. We speculated that the RT-RPA step possibly exerts an inhibitory effect on the T7-CRISPR/Cas13a step, or/and vice versa, which could involve inter-reaction interference, among other possible inhibitory factors. For example, T7 transcription may compete with RPA for binding to the common dsDNA substrate (as addressed later). A widely applied method to tackle an inhibitor issue in a molecular assay is to dilute the inhibitors.^31,32^ Based on a similar idea, we tested the effect of diluting the potential inhibitory factors by adding water to a starting one-pot SHERLOCK formulation, which as mentioned above had simply combined the reaction ingredients from two-step SHERLOCK. Addition of water at several test volumes was consistently found to improve the assay activity (Figure 2). Among these volumes, which together demonstrated a dose-dependent response in the magnitudes of dilution effect, the median condition was identified as optimal, conferring the most dramatic improvement in signal amplification (Figure 2). This may represent the best balancing point between the dilution of inhibitory factors and the retention of sufficient reagent concentrations to support the detection reaction, considering that excessive dilution could lead to reduced assay activity.^32^ These results are thus consistent with the idea that inhibitory interaction(s) between different groups of enzymatic reactions may play a role in one-pot SHERLOCK and the effect can be alleviated by dilution. The dilution, however, involving all reaction components simultaneously, may impose collateral impact on the other groups of reactions while relieving the inhibitory effect from the target reaction of interest. Moreover, the dilution could also impair the activity of the target reaction itself. Thus, the capacity of the universal dilution strategy would be restricted by a window where the intended reduction in the inhibitory effect outweighs unintended loss of assay activity due to dilution. We anticipated that additional optimizations, designed to overcome an inhibitory interaction without the negative collateral effect, could further enhance the assay activity. It is noted that addition of water into the formulation was the very first step of all optimizations in this study. Any other optimizations were made following this step, including those for individual reagents against potential overdilution, among others.

### Intermediate Pool of RPA Amplicons Free from T7 Interference Sustains Maximal RPA Amplification

**Figure 3:**
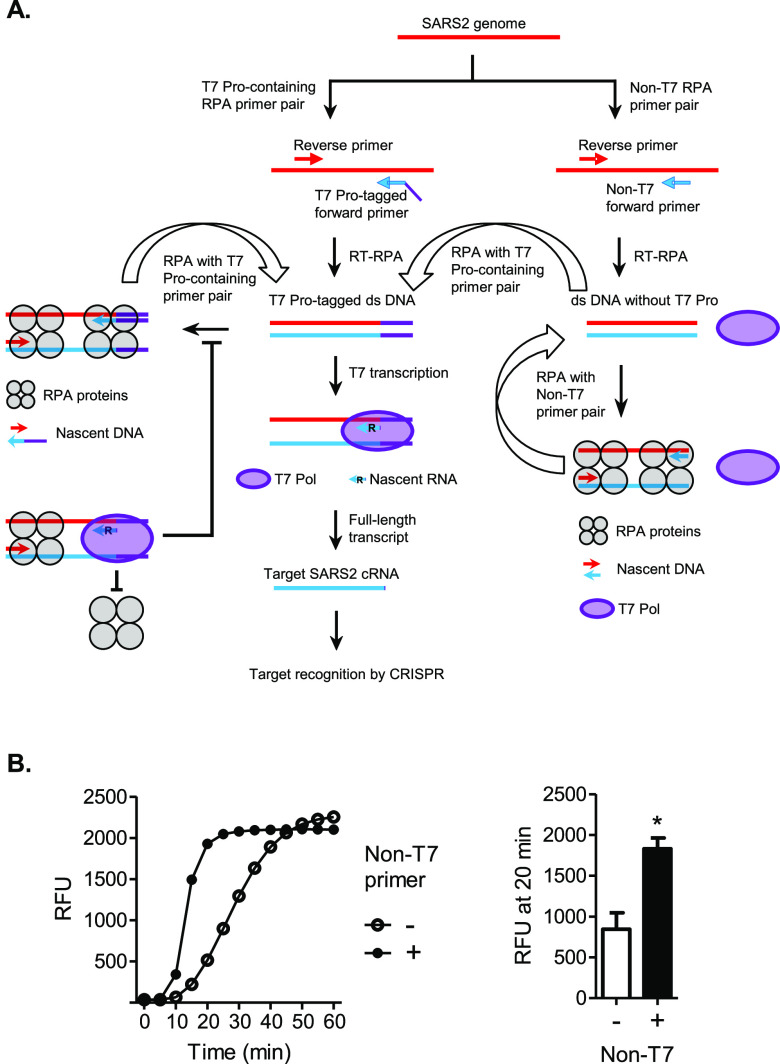
Adding an RPA forward primer without the T7 promoter (non-T7 forward primer) enhances one-pot SHERLOCK. (A) Molecular model: non-T7 primer frees RPA from T7 interference. In general, DNA amplicons generated from a previous round of RPA reaction become templates for the next round of reaction. Thus, continuous RPA cycles lead to exponential amplification. The productivity of RPA depends on new DNA strands to be effectively initiated and extended by RPA proteins from both ends of the template. However, in the scenario of RPA using the T7 promoter (T7 Pro)-tagged forward primer, which generates DNA amplicons bearing the T7 Pro sequence, T7 polymerase (T7 Pol) competes with RPA proteins for accessing the template from the T7-Pro end, limiting the full potential of RPA. The addition of a non-T7 promoter, however, leads to a pool of RPA amplicons without T7 Pro, which allows RPA cycles to operate at maximal capacity, free from T7 interference. To the DNA amplicons without T7 Pro, RPA can also introduce the T7 Pro-tagged forward primer (T7 Pro as an overhang portion of a primer), free from T7 interference as well, to produce DNA amplicons bearing the T7 Pro sequence. Thus, this RPA branch eventually moves the maximally amplified pool of DNA amplicons into the main flow of SHERLOCK. (B) Addition of non-T7 forward primer enhances one-pot SHERLOCK. SHERLOCK reactions were performed in the absence (−) or presence (+) of a non-T7 forward primer, with a SARS2 RNA concentration of 7.853 × 10^5^ cp/μL. Left: amplification plot shows RFU at indicated time points in the SHERLOCK reaction and represents three independent experiments showing similar patterns. Right: data were statistically analyzed, comparing the amplification signals at the time point of 20 min. The bar graph shows mean ± SEM. The significant difference in RFUs was determined by paired *t*-test: *p < 0.05.

In a SHERLOCK assay, RPA and T7 reactions share the same substrate (Figure 3A). The dsDNA amplicons produced by RPA become new templates to be occupied by RPA proteins in subsequent amplification cycles. The T7 promoter (T7 Pro) in these molecules, introduced through a T7 Pro-tagged forward RPA primer, however, also enables their binding by T7 polymerase (T7 Pol) for transcriptional amplification. In the traditional two-step SHERLOCK assay, the two amplification reactions are separated, allowing for fully independent and free access to and use of their dsDNA templates. However, when crowded together in a one-pot environment, they may become faced with a potential interference from competition with each other based on the need for the same template. This could disrupt the access to substrate, activation of amplification initiation, or progress of strand elongation.

To minimize this potentially inhibitory interaction, we introduced an RPA forward primer without the T7 promoter (non-T7 forward primer), which shares with the regular T7 Pro-tagged forward primer a common reverse primer, into the one-pot SHERLOCK assay. We anticipated that, using the non-T7 primer pair, RPA cycles are able to generate dsDNA amplicons that contain no T7 Pro sequence and keep using them as templates for maximal amplification free from T7 interference (Figure 3A). This maximally amplified, intermediate pool of amplicons then joins and fuels the main flow of SHERLOCK since RPA can use the regular T7 Pro-containing primer pair to convert and amplify the non-T7 amplicons into T7 Pro-containing amplicons. Experiments confirmed that adding the non-T7 forward primer significantly enhanced signal amplification (Figures 3B and S3). The effect was observed at different SARS-CoV-2 template concentrations (Figures 3B and S3). In addition, the ratio of the T7-tagged forward primer to the non-T7 forward primer was found to be optimal at 1:1 (Figure S4). The enhancing effect of the non-T7 forward primer supports the idea that freeing RPA from T7 interference through an intermediate pool of non-T7 DNA amplicons enhances one-pot SHERLOCK. Concerning the other arm of the interaction between RPA and T7 reactions, we have not established a strategy to specifically address the potential competitive interference with T7 by RPA. This is in spite of a possibility that non-T7 amplicons might partially divert away RPA enzymes, potentially reducing their competition against T7. Whether and to what extent that would benefit T7 or the assay overall remain unclear. However, we consider the potential interference with T7 by RPA a relatively minor issue, as elaborated in Results and Discussion S1.

### T7 Transcription Supplies crRNA to CRISPR through crDNA

**Figure 4:**
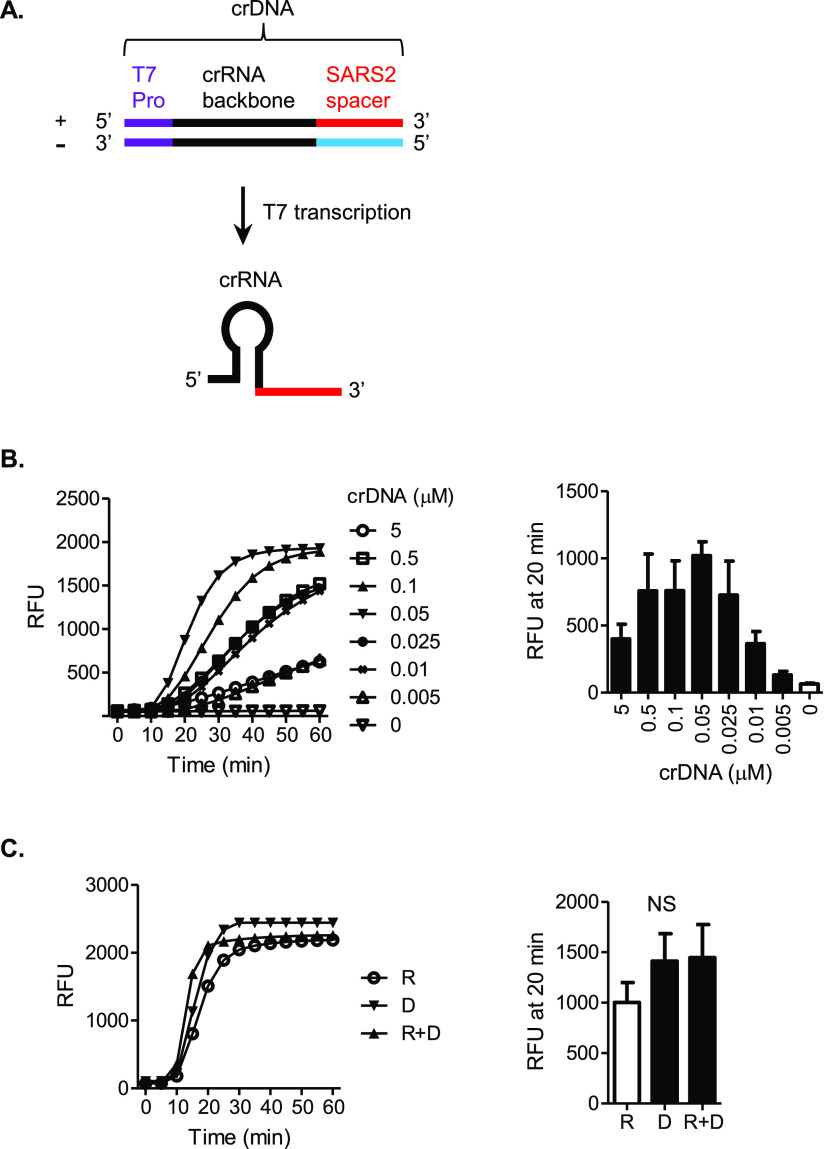
T7 transcription supplies crRNA to CRISPR through crDNA. (A) crDNA is a T7 promoter (T7 Pro)-tagged dsDNA that encodes a SARS-CoV-2 (SARS2)-targeting crRNA. SARS2-derived DNA or RNA sequences corresponding to the crRNA spacer are color-differentiated for strand polarities: red = positive sense (+), as in native SARS2 genomic RNA, and blue = negative sense (−), opposite to that of native SARS2 genomic RNA. For simplicity, T7 Pro and crRNA backbone sequences are indicated in single colors, purple and black, respectively, and not color-differentiated for strand polarities. T7 polymerase-catalyzed transcription produces a crRNA from the crDNA template. (B) Effect of crDNA concentration on one-pot SHERLOCK for SARS2. Stock solutions of crDNA at indicated concentrations were each tested (1.25 μL crDNA/80 μL reaction volume), replacing crRNA in the formulation. Left: amplification plot shows RFU at indicated time points in the SHERLOCK reaction and represents four independent experiments showing similar patterns. Right: bar graph compares amplification signals (mean ± SEM) between conditions with different crDNA stock concentrations. The 0.05 μM condition demonstrated the highest mean RFU value and was considered optimal. This concentration was used in the following experiments. (C) crDNA versus crRNA. SHERLOCK reactions were compared among conditions using crDNA alone, crRNA alone, or a combination of both. Left: amplification plot shows RFU at indicated time points in the SHERLOCK reaction and represents four independent experiments showing similar patterns. R = crRNA alone, D = crDNA alone, and R + D = combination of both crRNA and crDNA. Right: bar graph demonstrates the mean ± SEM of the amplification signals. No statistically significant difference was found among the three conditions. NS, not significant.

In the SHERLOCK assay, CRISPR activation requires two RNA components: a target RNA and a crRNA. T7 transcription provides the target RNA by using a T7 Pro-tagged dsDNA template. We asked whether the same mechanism could be utilized for the provision of crRNA, which would further connect T7 transcription and CRISPR reactions. Accordingly, we introduced a T7 Pro-tagged dsDNA template encoding the SARS-CoV-2-targeting crRNA, which we call a crDNA, into the one-pot SHERLOCK formulation, expecting that T7 transcription would readily use the crDNA template to synthesize the crRNA (Figure 4A). We tested several crDNA concentrations in the absence of crRNA in the one-pot SARS-CoV-2 SHERLOCK assay, which all resulted in signal amplification (Figure 4B). Under the optimal condition identified out of these concentrations, crDNA demonstrated a similar performance to crRNA or the combination of crDNA and crRNA (Figure 4C). These data indicate that crDNA can serve as a functional alternative to crRNA in the presence of T7 transcription. In the context of one-pot SHERLOCK, the crDNA strategy thus creates an additional positive interaction of T7 transcription with CRISPR. Furthermore, in a broader perspective, the in situ, real-time production and supply of crRNA from crDNA represents a practical advantage that can be taken in any CRISPR-based assays compatible with the T7 transcription. It is expected to reduce the challenge, complexity, and cost associated with RNA usage and enhance simplicity and user-friendliness.

### One-Pot SHERLOCK with CRISPR Targeting the Opposite Polarity of SARS-CoV-2 Genome Is Sensitive and Rapid

**Figure 5:**
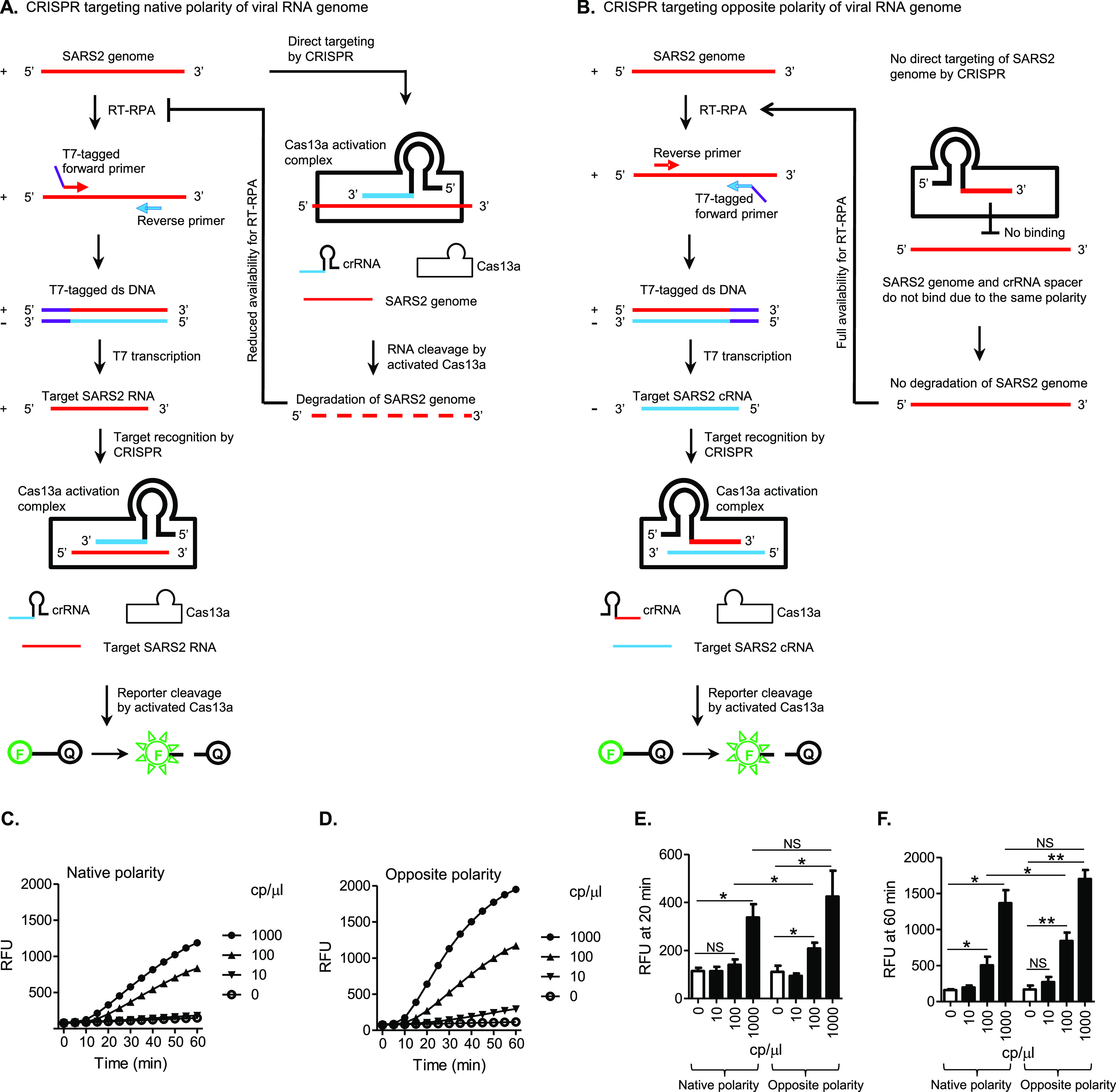
One-pot SHERLOCK with CRISPR targeting the opposite polarity of SARS-CoV-2 (SARS2) genome is sensitive and rapid. The format and coding in diagrams similar to those in Figure 1 are adopted here (see Figure 1 legends for details). (A) Traditional SHERLOCK design (SHERLOCK set ORF1ab), with CRISPR targeting the native polarity of the viral RNA genome. CRISPR is ready to directly recognize and degrade the input RNA templates as soon as the SHERLOCK reaction is set to start. This can deprive the RT of essential RNA substrates before it has converted sufficient amount of them into DNA molecules needed by RPA, especially when the input RNA concentration is at a low level, close to the detection limit. (B) New SHERLOCK design (SHERLOCK set opORF1ab), with CRISPR targeting the opposite polarity of the viral RNA genome. CRISPR does not recognize and degrade the input RNA due to polarity incompatibility and remains inactive, until encountering the cRNA target amplified by RT-RPA and T7 transcription. Thus, the input RNA amount is fully available for maximal amplification. (C,D) SHERLOCK reactions were tested for detection of SARS2 RNA at the indicated concentrations, based on CRISPR targeting the native polarity (C) and the opposite polarity (D) of the viral genome, respectively. Amplification plots show RFU at indicated time points in the SHERLOCK reactions and represent four independent experiments. (E,F) Data from (C,D) were statistically analyzed for difference in amplification signals between conditions at the time point of 20 min (E) or 60 min (F). NS = not significant, *p < 0.05 and **p < 0.01.

In a SHERLOCK assay, CRISPR can be directed at either the native polarity of the viral genome or the opposite polarity, and as described earlier, the opposite polarity-targeting CRISPR design affords a potentially higher assay specificity (Figures S1 and S2). We proceeded here to evaluate whether the polarity-targeting choice affects assay sensitivity. We predicted that in the scenario of CRISPR targeting the native polarity (the traditional design), the input SARS-CoV-2 genomic RNA templates are directly recognizable by CRISPR and are subject to degradation (at least partially). On the one hand, this could lead to a quick CRISPR detection if the concentration of RNA templates available is high enough for CRISPR to generate a detectable signal; on the other hand, however, this could deprive RT-RPA of needed RNA templates, rendering it ineffective, especially when the RNA templates are at a low concentration, close to the detection limit. It is important to note that CRISPR detection heavily depends on the molecular amplification by RT-RPA while not sensitive enough by itself. Therefore, the competition between CRISPR and RT-RPA for the common RNA substrate places a limit on the assay sensitivity, particularly in dealing with low target concentrations (Figure 5A). In contrast, CRISPR targeting the opposite polarity (the current strategy) does not allow direct recognition and degradation of the input viral RNA templates (with the native polarity) by CRISPR. Thus, they are fully available to RT-RPA for maximum amplification. This is expected to best fulfill the high-sensitivity detection capacity of SHERLOCK, which depends on all the three amplification mechanisms, RT-RPA, followed by T7 and CRISPR (Figure 5B). To test these predictions, we performed one-pot SARS-CoV-2 SHERLOCK comparing the two polarity-targeting strategies using the SHERLOCK sets ORF1ab and opORF1ab (Figure 5C–F). These experiments were based on the optimized one-pot SHERLOCK formulation (components detailed in Materials and Methods). At 1000 copies (cp)/μL SARS-CoV-2 RNA, both strategies showed no significant difference in signal amplification patterns, with a trend of stronger signals in opposite-polarity targeting. At 100 cp/μL SARS-CoV-2 RNA, however, opposite-polarity targeting led to significantly faster and stronger signal amplification than native-polarity targeting. In particular, opposite-polarity targeting demonstrated significant detection at the early, 20 min time point, while native-polarity targeting failed to. When SARS-CoV-2 RNA dropped to a 10 cp/μL concentration, neither strategy generated a significant detection signal (Figure 5C–F). These data indicate that the opposite polarity-targeting CRISPR strategy, which avoids the competition between CRISPR and RT-RPA, enhances the sensitivity and speed of one-pot SHERLOCK at a low target concentration close to the detection limit. Notably, in addition, the assay performance achieved here meets the sensitivity requirement for population SARS-CoV-2 screening test, which was estimated to be 100 cp/μL.^33^

The optimized one-pot SHERLOCK formulation covered the major optimization strategies addressing interactions between different enzymes/reactions (Figures 1 through 5) and integrated additional optimizations including traditional-way optimizations addressing individual enzymatic reactions. These additional optimizations are described in Results and Discussion S1 and Figures S5 through S11. Finally, using several different SHERLOCK sets, we confirmed that the major optimization strategies that address interactions between different enzymes/reactions function in a sequence-independent manner (Results and Discussion S1 and Figures S2 and S12).

## Conclusions

This study raised novel opportunities in the development of one-pot SHERLOCK diagnostics, which integrate isothermal (37 °C) RT, RPA, T7, and CRISPR/Cas13a reactions in a single formulation. Several optimization strategies were found to significantly enhance assay performance by addressing, in an environment crowding a large number of enzymes, interactions among different reaction types—a new layer of complicating factors largely untouched in previous assay optimization studies. These in combination with a few traditional-type optimizations contributed to a specific, sensitive, and rapid one-pot SHERLOCK assay for SARS-CoV-2 detection. High specificity was verified with a panel of negative control coronaviruses/respiratory viruses including those most closely related to SARS-CoV-2. The assay demonstrated an effective detection of SARS-CoV-2 RNA at 100 cp/μL within 20 min. This is about 100 times more sensitive than a rapid antigen test and meets the estimated requirement by large-scale SARS-CoV-2 screening.

The one-pot SHERLOCK assay in its current form, however, is not yet considered an ultimate end product having reached the fullness of its capacity. Designed on a proof-of-principle basis, the study represents an initial exploration of the feasibility to improve such a complex diagnostic system as one-pot SHERLOCK by targeting multi-reaction interactions. While the data presented here have fulfilled that scope, the network of interactions among the SHERLOCK enzymes, the effects of these interactions on the assay, and the strategies to resolve negative interactions and harness positive interactions remain to be fully studied. Advancements are anticipated toward 1 cp/μL sensitivity as typically seen in two-step SHERLOCK and PCR assays.

Nevertheless, this study has laid a foundation that would encourage and facilitate such advancements. The strategies for optimizing multi-enzyme compatibility and coordination identified in this study are based on common molecular biology principles and are not restricted to any specific buffer conditions. Thus, they could be readily adopted by other SHERLOCK assays such as the SHINE assays and enhance their activity. Their underlying general rationale should also be applicable to the development of additional new optimization strategies.

## Supporting Information Available

Additional texts, detailed sequence information of nucleic acids involved in this study, and additional experimental details and data (PDF)
