# Supplementary material for: Strategies to Improve Multi-enzyme Compatibility and Coordination in One-Pot SHERLOCK
Source: Anal Chem. 2023 Jun 30;95(28):10522–31. doi: 10.1021/acs.analchem.2c05032 (PMC10357406; doi:10.1021/acs.analchem.2c05032)
Supplement: Supplementary file 1 — ac2c05032_si_001.pdf [file ac2c05032_si_001.pdf]

Supporting information

Strategies to improve multi-enzyme compatibility and coordination in one-pot SHERLOCK

Hongzhao Li[1], Dominic M S Kielich[2], Guodong Liu[3], Greg Smith[1], Alexander Bello[3], James E Strong[2,3,4], and Bradley S Pickering[1,2,5*]

[1]National Centre for Foreign Animal Disease, Canadian Food Inspection Agency, Winnipeg, Manitoba R3E 3M4, Canada
[2]Department of Medical Microbiology and Infectious Diseases, College of Medicine, Faculty of Health Sciences, University of Manitoba, Winnipeg, Manitoba R3E 0J9, Canada;
[3]National Microbiology Laboratory, Public Health Agency of Canada, Winnipeg, Manitoba R3E 3M4, Canada;
[4]Department of Pediatrics & Child Health, College of Medicine, Faculty of Health Sciences, University of Manitoba, Winnipeg, Manitoba R3A 1S1, Canada;
[5]Iowa State University, College of Veterinary Medicine, Department of Veterinary Microbiology and Preventive Medicine, Ames Iowa 50011, United States of America

* Correspondence to: bradley.pickering@inspection.gc.ca

Table of contents

Introduction S1 ……………………………………………………………………………S-3

Materials and methods S1……………………………………………………………………S-4

Results and discussion S1……………………………………………………………………S-7

Table S1. Primer and crRNA/crDNA sets (SHERLOCK sets) and fluorescent RNA reporters……………………………………………………………………………………..S-12

Figure S1. Hypothetical model: Directing CRISPR at the opposite polarity of the viral genome in one-pot SHERLOCK may offer enhanced capacity of testing specificity. ……………………S-13

Figure S2. A SHERLOCK set targeting the opposite polarity of the SARS-CoV-2 (SARS2) genome has improved specificity over its counterpart set targeting the native polarity. …………..…..S-14

Figure S3. Addition of non-T7 forward primer enhances one-pot SHERLOCK with a lower template concentration…………………………………………... ……………………S-15

Figure S4. Effect of the ratio of T7-tagged forward primer to non-T7 forward primer ………S-16

Figure S5. Optimization of reverse transcription (RT) to facilitate RPA ………………………S-17

Figure S6. Effect of the protease buffer on one-pot SHERLOCK ………………………….………S-18

Figure S7. Effect of TCEP on one-pot SHERLOCK ……………………………………..………S-19

Figure S8. Effect of the RPA primer concentrations on one-pot SHERLOCK.…………………S-20

Figure S9. Effect of MgCl2 concentration on one-pot SHERLOCK..…………………...………S-21
Figure S10. Effect of Cas13a concentration on one-pot SHERLOCK..………………...……S-22
Figure S11. Effect of the fluorescent CRISPR reporter type on one-pot SHERLOCK..………S-23
Figure S12. Optimizations are not sequence-specific………………..………………...…...…S-24
References…………………………………………………..………………...………S-25

Introduction S1

Limitations of PCR-based diagnostics for infectious diseases

Accessible, rapid diagnostics for infectious diseases are critical for responsive public health measures to mitigate the spread of infections and provide timely patient care.[1] This is well highlighted by the coronavirus disease 2019 (COVID-19) pandemic, driven by severe acute respiratory syndrome coronavirus 2 (SARS-CoV-2).[2] Quantitative polymerase chain reaction (qPCR) remains the gold standard diagnostic test for early-stage infections, characterized by highly sensitive and specific nucleic acid detection.[3,4] However, PCR-based diagnostics, dependent on thermal cycling involving high temperatures (e.g. 95 °C), require specialized expertise, sophisticated equipment and an ample power source. As a result, these applications have limited availability, which are largely delivered from centralized laboratories in resource-abundant settings and have generally slow turnaround times. Their accessibility and capacity can be overwhelmed by high demands for widespread testing in a fast spreading outbreak, such as the Omicron surge of the COVID-19 pandemic.[3-5]

Additional advantages of SHERLOCK

SHERLOCK has additional, considerable advantages relative to Cas12-based CRISPR diagnostics. It has an extra molecular amplification mechanism based on T7-mediated transcriptional amplification, potentially conferring an enhanced capacity for sensitive detection. In addition, it does not require a protospacer adjacent motif (PAM) in the target sequence, which is often needed in target recognition by Cas12.[6] Therefore, this allows more flexibility in designing sequences to be targeted by CRISPR.

SHERLOCK is highly customizable and has a fast turnaround time for designing a diagnostic test against new pathogens. While all the other components in a SHERLOCK formulation are established and available, new primers and crRNAs can be designed in less than a week.[7] It is anticipated that within a matter of weeks a SHERLOCK assay can be deployed against an emerging pathogen after its genomic sequences (even if partially) are available. This is in contrast to rapid antigen tests, where it takes months to generate the required antibodies for antigen detection. Therefore, SHERLOCK has the advantage over rapid antigen tests in quick response to outbreaks of new pathogens (or pathogen variants with distinct antigenicity that evades antibody-armed detection) coupled with its higher sensitivity and specificity.[8]

Overview of optimizations targeting multi-enzyme interactions

The strategies included, among others: the directing of CRISPR at a T7 RNA transcript with strand polarity opposite to that of the native viral genome, to avoid early degradation of input RNA templates by CRISPR and conserve their full availability as substrates for maximal RT-RPA amplification; the addition of a forward RPA primer without the T7 promoter tag to create an intermediate pool of RPA amplicons that sustain maximal RPA amplification free from T7 interference; the enhancement of RT enzyme activities for the production of complementary DNA (cDNA) strand free from DNA-RNA hybrids to facilitate quick start and progression of RPA; and the introduction of crDNA, a template used by T7 transcription to supply crRNA for Cas13a.

Materials and Methods S1

Biosafety statement

All experiments involving infectious SARS-CoV-2, SARS-CoV or MERS-CoV were conducted in containment level 3 Zoonotic (SARS-CoV-2 and MERS-CoV) and containment level 4 (SARS-CoV) laboratories, respectively, at the National Centre for Foreign Animal Diseases (NCFAD), Canadian Food Inspection Agency (CFIA), following the institutional standard operating procedures.

Cell culture-derived viral RNAs

SARS-CoV-2, SARS-CoV and MERS-CoV propagated in Vero E6 cell culture were inactivated by the TriPure Isolation Reagent (Millipore Sigma, 11667165001) by mixing 100 µl culture supernatants with 900 µl TriPure in a 1.5 ml Eppendorf tube. TriPure-inactivated samples were then extracted for viral RNA using RNeasy Mini Kit (Qiagen, 74104) or the MagMAX CORE Nucleic Acid Purification Kit (ThermoFisher Scientific, A32700) on a KingFisher magnetic particle processor (ThermoFisher Scientific, A31508). Archived RNA samples were directly available from NCFAD for other coronaviruses, as well as influenza viruses. The other coronaviruses included infectious bronchitis virus (IBV), transmissible gastroenteritis virus (TGEV) and porcine epidemic diarrhea virus (PEDV). The influenza viruses included the H1N1 and H7N9 strains. RNA concentrations were quantified on a NanoDrop One spectrophotometer (ThermoFisher Scientific, ND-ONE-W4). These viral RNAs were used at a final concentration of 12.5 pg/µl for specificity testing of the SARS-CoV-2 SHERLOCK assay, except that in Figure S2 (see below) SARS-CoV RNA was used at 1 ng/µl, or 6.312×10^7 cp/µl. During the assay sensitivity optimization process, SARS-CoV-2 RNA was used at final concentrations ranging from 12.5 pg/µl, or 7.853×10^5 cp/µl, down to 1 cp/µl.

Sequences of RT-RPA primers, CRISPR RNAs (crRNAs), CRISPR DNAs (crDNAs) and RNA reporters

A set of RPA primers and crRNA (SHERLOCK set), targeting sequence locations in the ORF1ab region of the SARS-CoV-2 genome, were previously published by Feng Zhang's group.[9] Based on those target sequence locations, two SHERLOCK sets were designed in this study, aimed at different polarities of target RNA sequences and named as SHERLOCK set ORF1ab and SHERLOCK set opORF1ab, respectively (Table S1). In the ORF1ab set, T7 transcription amplifies an RNA transcript with the native polarity as the SARS-CoV-2 genome (positive sense), which is then recognized by a crRNA harboring a spacer sequence of negative sense (opposite polarity relative to native SARS-CoV-2 genome). The ORF1ab set is consistent with the traditional design in targeting the native polarity of the viral RNA genome, and has exactly the same primer sequences and crRNA sequence as those by Feng Zhang's group. In the opORF1ab set, T7 transcription generates an RNA strand of opposite polarity relative to the native SARS-CoV-2 genome (negative sense), which is then targeted by a crRNA with a positive-sense spacer. Six additional SARS-CoV-2 SHERLOCK sets were similarly designed, targeting the E or S gene (Table S1). Among these, the S1 set and S2/opS2 sets contained sequences derived from previous publications.[9,10] Note that the opORF1ab set was the default SHERLOCK set used throughout the optimization experiments unless any other SHERLOCK set was specified. In some of the SHERLOCK sets (Table S1), we also tested the use of a "crDNA", a T7 promoter-tagged double-stranded DNA template encoding a crRNA. A crDNA was first synthesized as single-stranded

DNA oligos and then annealed into a double-stranded DNA. For Cas13a-based detection, four types of fluorescent RNA reporters were tested (Table S1).

Base one-pot SHERLOCK formulation and targets of optimization

A 50-µl reaction included the following reagents: one TwistAmp pellet, 29.5 µl TwistAmp rehydration buffer, 3.445 µl H2O, 0.48 µl forward and reverse RPA primer mix (50 uM each primer), 1.25 µl RNase inhibitor (40 U/µl), 2.5 µl RevertAid reverse transcriptase (200 U/µl), 2.5 µl TwistAmp magnesium acetate (280 mM), 0.45 µl MgCl2 (1 M), 2 µl rNTP mix (25 mM each), 1.25 µl T7 RNA polymerase (50 U/µl), 1.25 µl CRISPR RNA (crRNA) (100 ng/µl), 3.125 µl RNase Alert v2 (2 µM in 1× RNase Alert buffer), 1.25 µl LwaCas13a protein (126.6 ng/µl in LwaCas13a storage buffer) and 1 µl SARS-CoV-2 target RNA or control sample. The name and commercial source of stock reagents used in these formulations (supplier, catalogue number) included: TwistAmp Basic RPA kit (TwistDx, TABAS03KIT), UltraPure DEPC-Treated Water (ThermoFisher Scientific, 750024), RNase inhibitor (New England BioLabs, M0314L), RevertAid reverse transcriptase (ThermoFisher Scientific, EP0442), $MgCl_2$ (ThermoFisher Scientific, AM9530G), rNTP mix (New England BioLabs, N0466L), NxGen T7 RNA Polymerase (Lucigen, 30223-1) and RNase Alert v2 (ThermoFisher Scientific, 4479768). In-house production of LwaCas13a (*Leptotrichia wadei* Cas13a) and its storage buffer was previously described.[11] During optimizations, all the reagents listed in the starting one-pot formulation or their alternatives were tested for the effect of their presence at varying concentrations or absence, except that the following had been kept constant: TwistAmp pellet, TwistAmp rehydration buffer, RNase inhibitor, TwistAmp magnesium acetate (280 mM), rNTP mix (25 mM each) and T7 RNA polymerase. Several reagents not listed in the starting formulation were also evaluated. Among these was a Qiagen protease buffer (Website: https://www.qiagen.com/us/products/discovery-and-translational-research/lab-essentials/enzymes/qiagen-protease-and-proteinase-k/, last accessed on 2023-04-21), which was considered a candidate sample lysis buffer, to be potentially employed in our future development of extraction-free SHERLOCK diagnostics. To make this buffer (pH 8.0, 10 mM Tris·Cl, 50 mM KCl, 1.5 mM MgCl2, 0.45% Tween 20 and 0.5% Triton X-100), the name and catalog number of stock solutions, available from ThermoFisher Scientific, included: Tris (AM9855G), KCl (AM9855G), MgCl2 (AM9530G), Tween 20 (28320) and TritonX-100 (85111). Other commercial reagents involved in the optimization were RNase H (New England BioLabs, M0297L), SuperScript™ IV Reverse Transcriptase (ThermoFisher Scientific, 18090010), Maxima H Minus Reverse Transcriptase (ThermoFisher Scientific, EP0751), Maxima Reverse Transcriptase (ThermoFisher Scientific, EP0742), M-MuLV Reverse Transcriptase (New England BioLabs, M0253L) and Tris(2-carboxyethyl)phosphine hydrochloride solution, or TCEP (Millipore Sigma, 646547).

The running condition of one-pot SHERLOCK reactions

This remained the same throughout the optimization experiments. The reaction was first assembled on ice and then incubated at 37 °C. Fluorescent signals were acquired using the FAM channel on an ESEQuant TS2.4 real-time fluorometer (Qiagen, ESEQuant TS2.4) or on a CFX96 Real-Time PCR System (Bio-Rad).

Statistical analysis

Significance of difference in the fluorescent intensities generated in the SHERLOCK assay was calculated by *Student's* paired *t* test in GraphPad Prism. A p value less than 0.05 was defined as significant.

Results and discussion S1

Directing CRISPR at the opposite polarity of the viral genome in one-pot SHERLOCK may offer enhanced capacity of testing specificity and ease of crRNA sequence selection

In SHERLOCK design, CRISPR can be directed at either the native or opposite polarity of the target viral genome (Figure S1). The prevalence of native-polarity targeting in traditional assays might be explained by an "ease of process". As a quick, early step in designing a set of RPA primers and their matching crRNA (a SHERLOCK set), a primer sequence may be directly copied from the original SARS-CoV-2 genomic sequence (available in native polarity from public databases), and a standard T7 Pro sequence can be simply added to its 5' end to form the T7 Pro-tagged forward primer (Figure S1A). Another possible explanation might be the consideration that, by targeting the native SARS-CoV-2 polarity, CRISPR could use both T7-transcribed target RNA and the original input viral RNA to generate detection signals. If the input viral RNA is at a high enough concentration, which has not been considerably consumed by RT, a rapid detection could be possible, through direct activation of CRISPR without the need for RT-RPA and T7 transcription to first amplify the target molecules to a concentration level detectable by CRISPR. However, in this situation, the specificity of the assay is determined only by the crRNA but not contributed to by the RPA primers (Figure S1A). Some recent studies suggested that CRISPR/Cas13a detection can have some extent of tolerance to mismatches between the spacer and target sequences,[11,12] although in another case variants differing by one nucleotide could be distinguished, implying a mismatch restriction.[7] In theory mismatch tolerance by CRISPR raises the possibility of false positive results when the assay specificity relies on only the crRNA. In a hypothetical model concerning assay specificity, for example, a SARS-CoV-2-related virus (e.g. SARS-CoV or MERS-CoV), present at a high concentration and containing a genomic sequence similar to that targeted by the crRNA of a SARS-CoV-2 SHERLOCK assay, could directly activate CRISPR (Figure S1A). Such potential false positive results could be reduced by choosing a CRISPR target sequence distinct from any sequence found in viruses that are closely related to SARS-CoV-2 but at the same time conserved among SARS-CoV-2 variants to maintain assay sensitivity. The high-stringency criteria, however, also means low flexibility in sequence selection during CRISPR design. In contrast to the traditional native polarity-targeting CRISPR design, if CRISPR is directed at the opposite polarity of the viral genome, the specificity of the entire SHERLOCK assay would be determined by both the crRNA and RPA primers together (Figure S1B). This is an enhanced, two-layered specificity, restricted by three sequences and two types of reactions. Under this design format, the stringency in crRNA target sequence selection could be relatively relaxed due to the reinforcement by RPA primers.

This hypothetical model was tested experimentally (Figure S2). SARS-CoV-2 SHERLOCK sets E1 and opE1 (Table S1) target the native and opposite polarities of the SARS-CoV-2 genome, respectively, but share the same genomic locations recognized by RT-RPA primers and crRNAs. In these SHERLOCK sets, the RT-RPA primers have multiple mismatches with SARS-CoV including those at the 3' end of the primers. Thus, these primers were predicted to be specific for SARS-CoV-2 and unable to mediate RPA amplification of SARS-CoV sequences.[13,14] The spacer sequences of the crRNAs, E1-Cr (targeting the native polarity) and opE1-Cr (targeting the opposite polarity), have one mismatch with the counterpart region of SARS-CoV (Figure S2A). E1-Cr in the absence of RT-RPA primers mediated direct CRISPR/Cas13a activation by SARS-CoV, while in contrast opE1-Cr failed to (Figure S2B). On the level of the entire SHERLOCK sets, the E1 set

detected both SARS-CoV-2 and, non-specifically, SARS-CoV, whereas the opE1 set detected only SARS-CoV-2 but not SARS-CoV (Figure S2C). These data provide an example that direct CRISPR/Cas13a activation bypassing RT-RPA could lead to non-specific target recognition in a native polarity-targeting SHERLOCK set (as illustrated in Figure S1A); however, the non-specific target recognition could be eliminated in an opposite polarity-targeting SHERLOCK set by the requirement of RPA pre-amplification with two specific primers, in combination with the polarity incompatibility between the crRNA and native viral RNA template (as illustrated in Figure S1B).

It should be noted, however, that the polarity effect on specificity is expected to be template concentration-dependent. In traditional SHERLOCK assays, CRISPR detection based on LwaCas13a needs to be coupled with a pre-amplification step to achieve high sensitivity. Direct activation of CRISPR/LwaCas13a to generate detectable amplification signal would require a high template concentration.[7,15] However, the more recently reported LbuCas13a (*Leptotrichia buccalis* Cas13a) has demonstrated dramatically more robust activity, with potential to detect low template concentration at around 100 cp/µl level without pre-amplification.[15] We anticipate that in future development the incorporation of LbuCas13a into one-pot SHERLOCK will significantly enhance the assay sensitivity. In this context, the effect of direct CRISPR activation by RNA template with native polarity should be a key consideration.

It remains poorly understood how specific types of mismatches between the crRNA and template RNA affect CRISPR detection. The effect of mismatches might depend on their number, nucleotide types, locations within the spacer and the context of their neighboring sequences.[11] Currently, in native polarity-targeting SHERLOCK there is no consensus in crRNA design on what mismatches are needed in order to distinguish a specific target from a closely-related non-specific target, such as SARS-CoV-2 from SARS-CoV. Intuitively, the spacer sequence of a SARS-CoV-2 crRNA should contain a substantial (or maximal) degree of mismatches against SARS-CoV. On the other hand, the spacer sequence needs to allow the detection of SARS-CoV-2 variants in order to maintain assay sensitivity. Therefore, the spacer sequence is required to arise from the intersection of two sets of sequences, one set being evolutionarily variable between the closely related viruses SARS-CoV-2 and SARS-CoV and the other conserved among different variants of the SARS-CoV-2 virus. The restricted flexibility in sequence selection could pose a challenge to the design and screening of crRNAs since a large number of candidate sequences need to be tested to find a highly active crRNA.[7] In an opposite polarity-targeting SARS-CoV-2 SHERLOCK assay, the specificity of template recognition by crRNA against SARS-CoV is achievable on the level of polarity incompatibility, independently of dealing with the challenge of specific sequence selection (Figure S1B). Furthermore, in an opposite polarity-targeting SHERLOCK assay the specificity is guarded by three sequence "checkpoints", the RPA primers and crRNA, and can be conferred by any one of the three sequences (Figures S1B). This is in contrast to native-polarity targeting, where a non-specific crRNA recognition alone could deprive the entire SHERLOCK set of specificity by bypassing the function of RPA primers (Figure S1A).

Coupled with the crRNA sequence, RT-RPA primer sequences are the other key determinant of assay specificity. The effects of sequence mismatches on RT and RPA reactions have been relatively better understood than those on CRISPR/Cas13a. In RT, most mismatches between the primer and RNA template are extended to high extents even when they are located to the 3' end.[16-18] Therefore, a contribution of RT primer design to specificity optimization is negligible. In RPA,

mismatches between the primers and DNA template have been shown to have a variable inhibitory effect depending on the positions and nucleotide types of mismatches.[13,14] Certain types of mismatches adjacent to the 3' terminus demonstrated the most detrimental effect on the RPA reaction, such as terminal C-C and G-A mismatches. However, terminal A-C and T-G mismatches were well tolerated. The effects of these mismatches appear to bear similarity to those observed in PCR.[19] In addition, the combination of a terminal C-A mismatch and a penultimate C-C mismatch led to complete RPA reaction inhibition.[14] Another key parameter in RPA design is the size of primers. Unlike the crRNA spacer sequences for LwaCas13a, which have been constantly used at 28 nucleotides (nt) in length,[7,9-11] RPA primers reportedly range from 30 – 35 nt down to18 nt.[20,21] This appears to allow great flexibility in locating and adjusting primer target regions on the viral genome. Collectively, the available knowledge and known advantages in utilizing RPA primers encourage the idea of leveraging RPA primer design to maximize SHERLOCK assay specificity (a multi-checkpoint specificity).

In summary, the current knowledge together with the data presented above suggests that in designing a specific SHERLOCK assay the opposite polarity-targeting strategy may be a safer and easier choice over native polarity-targeting, with potentially enhanced capacity for specific detection. However, currently we would not draw definite conclusions on these aspects, with gaps of knowledge remaining to be filled by extensive future investigations, especially in how mismatches affect CRISPR/Cas13a detection. Instead, we present these possibilities to the field and encourage follow-up research that could potentially further advance the SHERLOCK diagnostic platform.

The potential interference with T7 by RPA

Although a competitive interaction can be intuitively perceivable as mutual between RPA and T7, we speculated that the interference with RPA may have a far greater impact on the assay activity than that with T7, based on two possible reasons. First, the amplification is exponential by RPA, the dominating target amplifier in SHERLOCK, but linear by T7. And second, RPA depends on the highly complex and delicate coordination among the activities of the recombinase (UvsX), recombinase loading factor (UvsY), single strand binding protein (SSB), DNA polymerase and other factors such as creatine kinase.[22] This may account for a sensitive and demanding nature of RPA and thus likely a susceptibility to disturbance. Consistent with this idea, a special, fine-tuned formulation to support optimal RPA activity is not available widely, except from the supplier company, TwistDx. In contrast, T7 transcription is a robust and easy assay, with a consistently stable performance in widespread applications based on various commercial and customized conditions. Taking these together, addressing the interference with RPA by T7 was considered here to be of higher priority, and progress has been made in that respect.

Optimization of activities in reverse transcription (RT) to remove hindrance to RPA

Previous studies found that RPA-based assays including those coupled with CRISPR have much lower sensitivity in detecting RNA than in detecting DNA. RNA detection requires an extra step, where a reverse transcriptase (RT enzyme) catalyzes the conversion of RNA into DNA (the substrate for RPA), which requires an RNA-dependent DNA polymerase domain, to synthesize the complementary DNA (cDNA) strand while an RNase H domain degrades the RNA template of the DNA-RNA hybrid duplex.[23] The remaining RNA strand attached to the cDNA was recently proposed to be a limiting factor for the subsequent RPA reaction.[24] Conceivably, it could cause a

block or delay in access to substrate, or the initiation or processivity of RPA. Increasing RNase H activity was thus believed to enhance RPA through enhanced removal of this RNA hindrance. However, the performance of the RT enzyme itself can be affected by interaction between its polymerase and RNase H activities. The RNase H activity may compete with the polymerase activity, limiting the length of cDNA synthesis or directly the polymerase activity.[25]

Related to these considerations, enzyme engineering has led to a diversification of RT enzymes with differential characteristics such as in RNase H activity, polymerase processivity, synthesis rate, forgiveness of template quality and quantity, or thermostability. In some cases, mutations in the RNase H domain that decrease the RNase H activity result in higher yield and longer cDNA products.[25] Taking these together, we further propose that optimizations that balance the polymerase and RNase H activities of the RT enzyme could provide an optimal condition for both removing the RNA obstacle to RPA and maintaining the overall functions of the RT enzyme. A net desired outcome would be the most efficient supply of cDNA templates from the RT to RPA reactions. We were then prompted to test a variety of RT enzymes with different polymerase activities and intrinsic RNase H activities, in the presence or absence of exogenous RNase H (Figure S5). Among these, the Moloney Murine Leukemia Virus RT enzyme (M-MuLV, MMLV) has wild-type polymerase and RNase H domains. In comparison, the other RT enzymes are all mutants of this wild-type version. RevertAid is known to have a significantly lower RNase H activity than that of Avian Myeloblastosis Virus (AMV) RT enzyme. SuperScript IV (SSIV) and Maxima H minus (Maxima H-) are characterized by high processivity and reduced (in SSIV) or no (in Maxima H-) RNase H activity. And finally, Maxima maintains both polymerase activity and RNase H activity and has increased synthesis rates compared to MMLV wild type. Titration experiments identified the optimal combination of enzyme types and concentrations, which included MMLV wild-type RT enzyme and exogenous RNase H and led to significantly enhanced signal amplification (Figure S5). Our results were consistent with the previous view that increasing RNase H activity enhances RPA, presumably through more effective degradation of the RNA template off the cDNA strand.[24] These data also suggest that changes in processivity or synthesis rate (functions directly related to the polymerase activity), at least those within the range of the differences among the enzymes tested here, do not have major effect on RT-RPA. This is probably due to the small size of typical RPA amplicons, slightly above 100 bp.

Additional optimizations

Since a field-deployable molecular test should not depend on RNA extraction which is required by current PCR diagnostics in centralized laboratories, we are developing an extraction-free sample processing method, based on a sample lysis buffer that supports the activity of a proteinase to degrade viral capsid and RNase proteins (for simplicity, proteinase buffer, or PB, see Materials and Methods S1 for formulation). Since the buffer does not contain any harsh chemical ingredients, we anticipated that transferring a considerably large volume of it (for maximum sample lysate input) into SHERLOCK would not impose an inhibitory effect. We not only confirmed this but also, fortuitously, found that it substantially enhanced the speed and strength of signal amplification at an optimal condition of 24.445 µl PB in a 80 µl total reaction volume (Figure S6). These results represent another positive interaction between reactions, and present a great potential for optimizations that coordinate sample processing and SHERLOCK detection. Moreover, while seeking inter-enzyme/reaction compatibility and coordination was given a particular emphasis as a new aspect of one-pot SHERLOCK development, the traditional-way optimizations, addressing

individual enzymatic reactions, were still considered fundamental here. A number of such optimizations identified factors with effect on the assay (Figures S7 through S11).

HUDSON (heating unextracted diagnostic samples to obliterate nucleases) is a promising extraction-free sample lysis method that has been coupled with SHERLOCK.[8] The HUDSON lysis buffer contains Tris (2-carboxyethyl) phosphine (TCEP), a reducing reagent, which was used at 100 mM in clinical sample treatment. Concerning the capacity of one-pot SHERLOCK in accommodating TCEP-containing sample lysates, we tested the addition of TCEP at 1, 5 and 10 mM final concentrations in the one-pot SHERLOCK formulation, equivalent to 1:100, 1:20 and 1:10 dilutions of sample lysates, respectively. While the 1 mM concentration did not show a significant effect, 5 and 10 mM substantially inhibited signal amplification (Figure S7). These results suggest that the use of TCEP for sample lysis could limit the capacity of sample input into one-pot SHERLOCK. This was also the rationale prompting us to seek an alternative way of sample lysis that could be accommodated by the core one-pot SHERLOCK formulation at a higher capacity, as just described above (Figure S6). In other experiments, we tested concentrations of RPA primers (Figure S8), enzyme cofactor Mg^{2+} (Figure S9) and Cas13a (Figure S10), as well as types of CRISPR reporters (Figure S11). All these new findings had been integrated into the optimized one-pot SHERLOCK formulation.

The optimizations are not sequence-specific

Using several additional SHERLOCK sets, targeting the E or S gene of SARS-CoV-2 (Table S1), we conducted confirmatory test of the major optimization strategies that address interactions between different enzymes/reactions (Figures S2 and S12). An opposite polarity-targeting SHERLOCK set (opE1) was found to be functional and specific, detecting SARS-CoV-2 but not SARS-CoV (Figure S2). In contrast, the native polarity-targeting counterpart (SHERLOCK set E1) detected SARS-CoV non-specifically (Figure S2). These results provide an example that opposite-polarity targeting confers higher capacity for assay specificity over native-polarity targeting. Furthermore, addition of water (Figure S12A, SHERLOCK set opE2) or introduction of a non-T7 forward primer (Figure S12B, SHERLOCK set opS1) improved signal amplification; a crDNA was functional and supported the SHERLOCK reaction similarly to its crRNA counterpart (Figure S12C, SHERLOCK set opE2); and opposite-polarity targeting showed stronger signal amplification than native-polarity targeting (Figure S12D, SHERLOCK set opS2). These data confirmed that the optimizations are not sequence-specific and work for optimizing assays that target other sequences.

Table S1. Primer and crRNA/crDNA sets (SHERLOCK sets) and fluorescent RNA reporters

SHERLOCK set or RNA reporter	SARS2 genomic target by SHERLOCK: gene, polarity	Type of SHERLOCK component	Name of SHERLOCK component	Sequence (5'-3')*
SHERLOCK set ORF1ab	ORF1ab, native	T7-tagged forw ard primer	T7-ORF1ab-F	GAAATTAATACGACTCACTATAGGGCGAAGTTGTAGGAGACATTATACTTAAACC
		Reverse primer	ORF1ab-R	TAGTAAGACTAGAATTGTCTACATAAGCAGC
		Non-T7 forw ard primer	ORF1ab-F	CGAAGTTGTAGGAGACATTATACTTAAACC
		crRNA	ORF1ab-Cr	GAUUUAGACUACCCCAAAAACGAAGGGGACUAAAACCCAACCUCUUCUGUAAUUUUUAAACUAU
SHERLOCK set opORF1ab	ORF1ab, opposite	T7-tagged forw ard primer	T7-opORF1ab-F	GAAATTAATACGACTCACTATAGGGTAGTAAGACTAGAATTGTCTACATAAGCAGC
		Reverse primer	opORF1ab-R	CGAAGTTGTAGGAGACATTATACTTAAACC
		Non-T7 forw ard primer	opORF1ab-F	TAGTAAGACTAGAATTGTCTACATAAGCAGC
		crRNA	opORF1ab-Cr	GAUUUAGACUACCCCAAAAACGAAGGGGACUAAAACAUAGUUUAAAAAUUACAGAAGAGGUUGG
		crDNA	T7opORF1abCrDNA	GAAATTAATACGACTCACTATAGGGGATTTAGACTACCCCAAAAACGAAGGGGACTAAAACATAGTTTAAAAATTACAGAAGAGGTTGG
SHERLOCK set E1	E, native	T7-tagged forw ard primer	T7-E1-F	GAAATTAATACGACTCACTATAGGGTTTGTAAGCACAAGCTGA
		Reverse primer	E1-R	AGAGTAAACGTAAAAAGAA
		crRNA	E1-Cr	GAUUUAGACUACCCCAAAAACGAAGGGGACUAAAACCUAGUGUAACUAGCAAGAAUACCACGAA
SHERLOCK set opE1	E, opposite	T7-tagged forw ard primer	T7-opE1-F	GAAATTAATACGACTCACTATAGGGAGAGTAAACGTAAAAAGAA
		Reverse primer	opE1-R	TTTGTAAGCACAAGCTGA
		crRNA	opE1-Cr	GAUUUAGACUACCCCAAAAACGAAGGGGACUAAAACUUCGUGGUAUUCUUGCUAGUUACACUAG
SHERLOCK set opE2	E, opposite	T7-tagged forw ard primer	T7-opE2-F	GAAATTAATACGACTCACTATAGGGCGTTAACAATATTGCAGCAGTACGCACACA
		Reverse primer	opE2-R	TCGTTTCGGAAGAGACAGGTACGTTAATAG
		Non-T7 forw ard primer	opE2-F	CGTTAACAATATTGCAGCAGTACGCACACA
		crRNA	opE2-Cr	GAUUUAGACUACCCCAAAAACGAAGGGGACUAAAACCUUCUUUUUCUUGCUUUCGUGGUAUUCU
		crDNA	T7opE2crDNA	GAAATTAATACGACTCACTATAGGGGATTTAGACTACCCCAAAAACGAAGGGGACTAAAACCTTCTTTTTCTTGCTTTCGTGGTATTCT
SHERLOCK set opS1	S, opposite	T7-tagged forw ard primer	T7-opS1-F	GAAATTAATACGACTCACTATAGGGTCCTAGGTTGAAGATAACCCACATAATAAG
		Reverse primer	opS1-R	AGGTTTCAAACTTTACTTGCTTTACATAGA
		Non-T7 forw ard primer	opS1-F	TCCTAGGTTGAAGATAACCCACATAATAAG
		crDNA	T7opS1crDNA	GAAATTAATACGACTCACTATAGGGGATTTAGACTACCCCAAAAACGAAGGGGACTAAAACCTTCTTCAGGTTGGACAGCTGGTGCTGC
SHERLOCK set S2	S, native	T7-tagged forw ard primer	T7-S2-F	GAAATTAATACGACTCACTATAGGGAGGTTTCAAACTTTACTTGCTTTACATAGA
		Reverse primer	S2-R	TCCTAGGTTGAAGATAACCCACATAATAAG
		Non-T7 forw ard primer	S2-F	AGGTTTCAAACTTTACTTGCTTTACATAGA
		crRNA	S2-Cr	GAUUUAGACUACCCCAAAAACGAAGGGGACUAAAACGCAGCACCAGCUGUCCAACCUGAAGAAG
SHERLOCK set opS2	S, opposite	T7-tagged forw ard primer	T7-opS2-F	GAAATTAATACGACTCACTATAGGGTCCTAGGTTGAAGATAACCCACATAATAAG
		Reverse primer	opS2-R	AGGTTTCAAACTTTACTTGCTTTACATAGA
		Non-T7 forw ard primer	opS2-F	TCCTAGGTTGAAGATAACCCACATAATAAG
		crRNA	opS2-Cr	GAUUUAGACUACCCCAAAAACGAAGGGGACUAAAACCUUCUUCAGGUUGGACAGCUGGUGCUGC
RNA reporter: RNase Alert v2	-	-	-	Commercial product (ThermoFisher Scientific, 4479768) w ithout sequence information
RNA reporter: U5	-	-	-	6-FAM/UUUUU/IABkFQ
RNA reporter: U7	-	-	-	6-FAM/UUUUUUU/IABkFQ
RNA reporter: U14	-	-	-	6-FAM/UUUUUUUUUUUUUU/IABkFQ

* Primers are single-stranded DNA, crRNAs and RNA reporters are single -stranded RNA, and crDNAs are double-stranded DNA. All sequences, except RNase Alert v2, w ere custom-synthesized by Integrated DNA Technologies.
-, Not applicable.

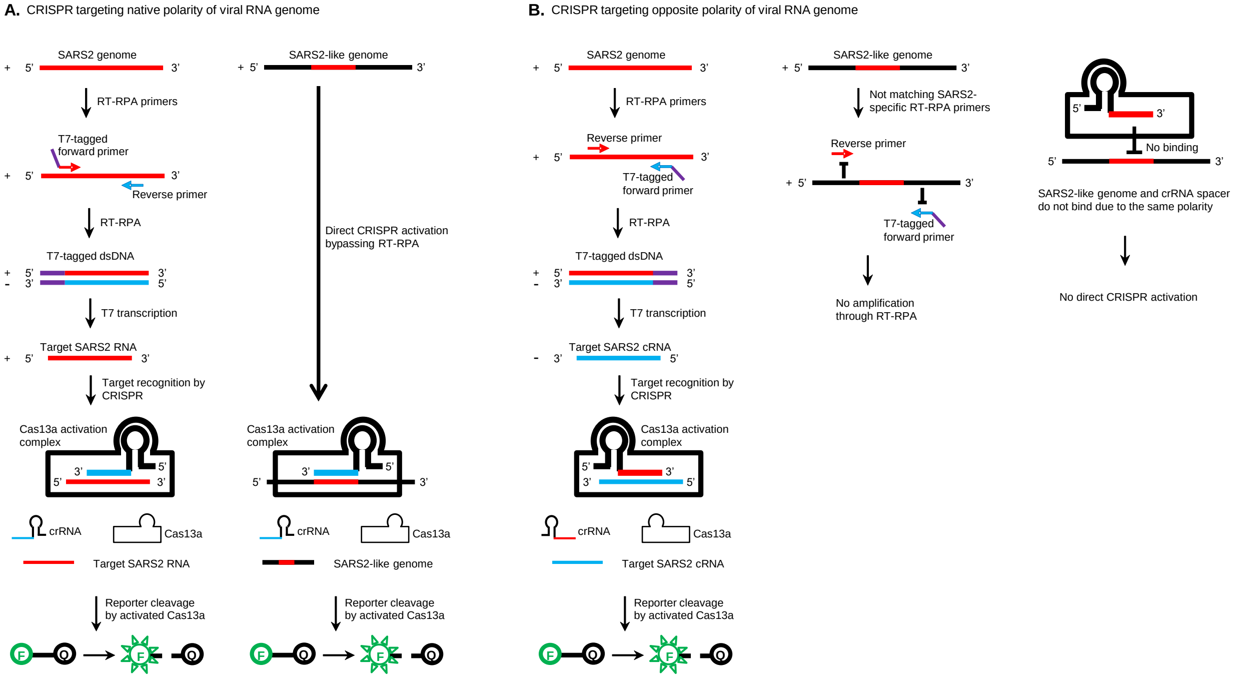

Figure S1. Hypothetical model: Directing CRISPR at the opposite polarity of the viral genome in one-pot SHERLOCK may offer enhanced capacity of testing specificity. Schematics are not drawn to scale or necessarily reflect the exact molecular shapes. The polarity of a SARS2 (or SARS2-like) sequence on an RNA or DNA strand is differentially indicated in red (native polarity = positive sense) versus blue (opposite polarity = negative sense). For simplicity, however, non-SARS2-like sequences are not color-differentiated for polarities, with both polarities shown in the same color. These sequences are represented in black, except that T7 promoter sequences are indicated in purple. In general, the polarity of an RNA or DNA strand, labelled as "+" (positive sense), or "-" (negative sense), was defined according to the polarity of the SARS2-like sequence it contains. The native SARS2 genomic sequence (positive-sense RNA strand, indicated in red) is first converted and amplified by RT-RPA into a T7 promoter-tagged double-stranded DNA (T7-tagged dsDNA). The DNA is then T7-transcribed and amplified into a final RNA strand for CRISPR targeting. The resulting activation of Cas13a leads to the cleavage of an RNA reporter and the release of fluorescent signals. In the RNA reporter, "F" and "Q" mean fluorophore and quencher, respectively. The final RNA strand to be targeted by CRISPR can have a native SARS2 genome polarity (+/red, as in panel A) or an opposite polarity (-/blue, as in panel B), depending on the polarity of the SARS2 sequence in the RPA primer that the T7 promoter sequence is attached to. Corresponding to that, the crRNA will have a complementary spacer sequence (-/blue in A or +/red in B), in order to bind the target RNA. As diagramed in parallel with the native SARS2 genome (the intended diagnostic target), a hypothetical SARS2-like viral genome (such as that from a related coronavirus) harbors a sequence (+/red) similar to the native SARS2 counterpart targeted by CRISPR, which could pose concern over assay specificity, depending on the SHERLOCK design strategy. **A.** Traditional SHERLOCK design (SHERLOCK set ORF1ab), with CRISPR targeting the native polarity of viral RNA genome. The hypothetical SARS2-like viral genome can directly activate CRISPR, without the dependence on specific pre-amplification conferred by RPA primers. **B.** New SHERKLOCK design (SHERLOCK set opORF1ab), with CRISPR targeting the opposite polarity of viral RNA genome. The crRNA only recognizes the target RNA transcript produced by RPA, which is a complementary version of the native SARS2 RNA genomic sequence (cRNA) with an opposite polarity. Concerning the hypothetical SARS2-like viral genome, it is not amplifiable by RPA to generate the cRNA target for CRISPR due to the lack of homologies to SARS2-specific RPA primers outside the crRNA target region. The unamplified viral genome is not recognizable by crRNA, thanks to incompatible polarities. In this scenario, the net assay specificity is collectively contributed to by both CRISPR and RPA, based on three SARS2-specific sequences (one from crRNA and two from RPA primers).

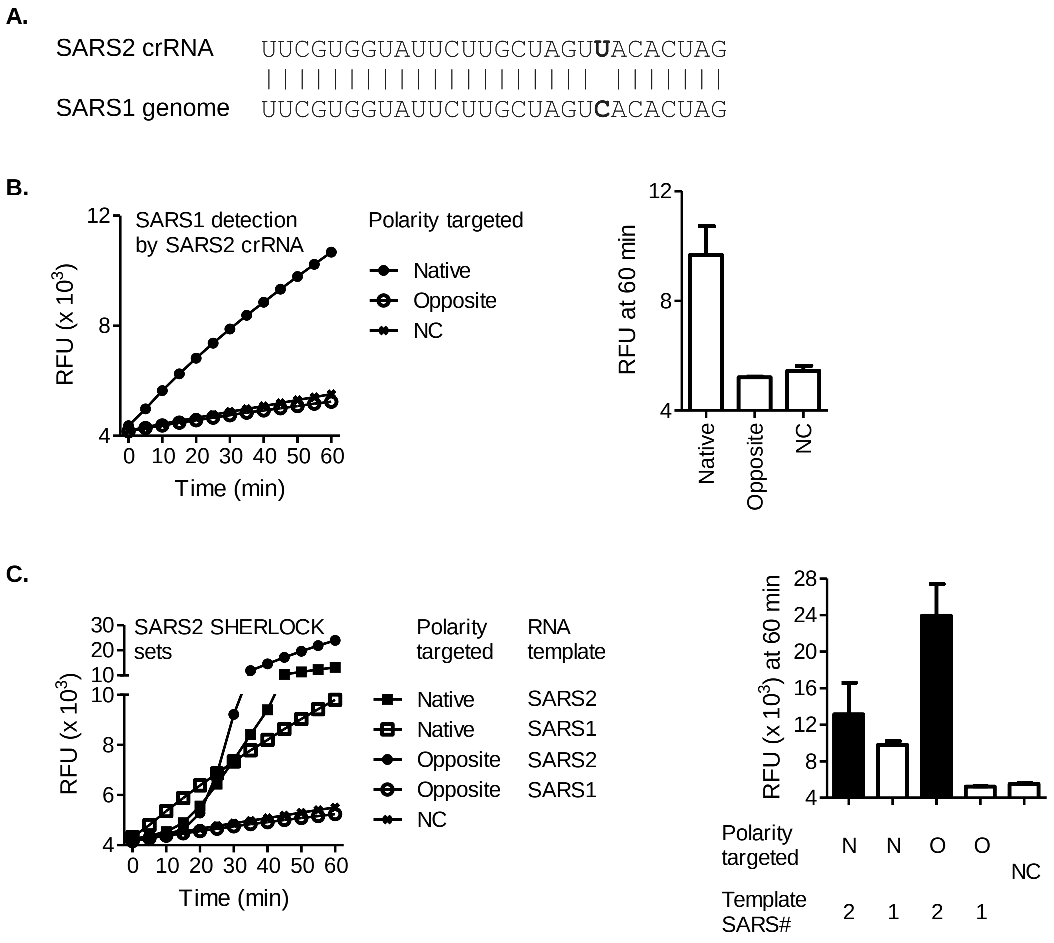

Figure S2. A SHERLOCK set targeting the opposite polarity of the SARS-CoV-2 (SARS2) genome has improved specificity over its counterpart set targeting the native polarity. A. SARS2 SHERLOCK sets E1 (native polarity-targeting) and opE1 (opposite polarity-targeting) (Table S1) have one mismatch in their spacer sequence with a SARS-CoV (SARS1) sequence. For simplicity a representative alignment is shown using a spacer sequence of native polarity. **B.** Test of direct CRISPR detection of SARS1 in the absence of RT-RPA pre-amplification. Reactions were performed using the optimized one-pot SHERLOCK formulation except that the RT-RPA primers were not added. SARS1 RNA concentration used was 6.312×10^{7} cp/µl. A SARS2 crRNA from either SHERLOCK set E1 (E1-Cr, native polarity-targeting) or from SHERLOCK set opE1 (opE1-Cr, opposite polarity-targeting) was involved in each reaction. NC, negative control without crRNA or template. Left: Amplification plot shows relative fluorescent units (RFU) at indicated time points in the SHERLOCK reaction, and represents three independent experiments showing similar patterns. Right: Bar graph indicates mean ± SEM of the three independent experiments for the time point of 60 min. **C.** Assay specificities were confirmed for entire SHERLOCK sets. The SARS2 SHERLOCK sets E1 and opE1 were tested for reactivity to SARS2 and SARS1 based on the optimized one-pot SHERLOCK formulation including RT-RPA primers. NC, negative control without crRNA or template. Left: Amplification plot shows relative fluorescent units (RFU) at indicated time points in the SHERLOCK reaction. Right: Bar graph indicates mean ± SEM of replicates for the time point of 60 min. N and O, native and opposite polarities, respectively. Numbers 2 and 1, SARS2 and SARS1, respectively.

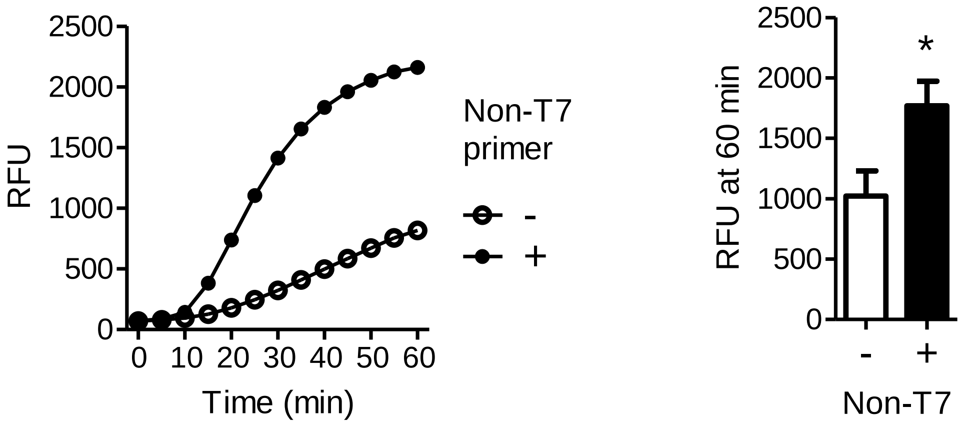

Figure S3. Addition of non-T7 forward primer enhances one-pot SHERLOCK with a lower template concentration. SHERLOCK reactions were performed in the absence (-) or presence (+) of non-T7 forward primer, with a SARS2 RNA concentration of 10^3 cp/µl. This is a repeat of Figure 3B, except that a SARS2 RNA concentration of 7.853×10^5 cp/µl was used in Figure 3B. Left: Amplification plot shows relative fluorescent units (RFU) at indicated time points in the SHERLOCK reaction, and represents four independent experiments showing similar patterns. Right: Data were statistically analyzed, comparing the amplification signals at the time point of 60 min. Bar graph shows mean ± SEM. Significant difference in RFUs was determined by paired *t* test: * $p < 0.05$.

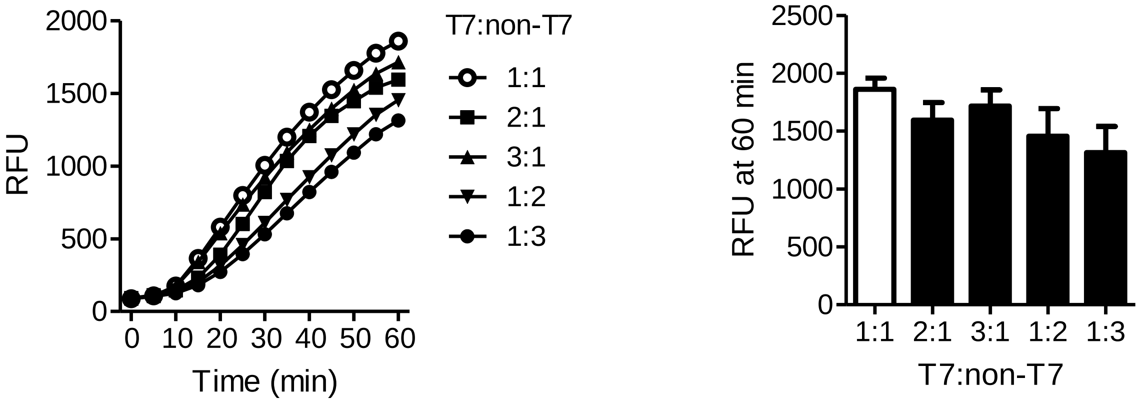

Figure S4. Effect of the ratio of T7-tagged forward primer to non-T7 forward primer. SHERLOCK reactions containing T7 promoter-tagged forward primer and non-T7 forward primer at each indicated ratio were tested, with a SARS2 RNA concentration of 10^3 cp/µl. Left: Amplification plot shows relative fluorescent units (RFU) at indicated time points in the SHERLOCK reaction, and represents two independent experiments showing similar patterns. Right: Bar graph indicates mean ± SEM of replicates for the time point of 60 min. The 1:1 ratio demonstrated the best performance and was used in the other experiments and included in the optimized SHERLOCK formulation. According to this condition, 1.8 µl RPA primer master mix (10 µM T7 promoter-tagged forward primer, 10 µM non-T7 forward primer and 20 µM reverse primer) was used in a 80 µl SHERLOCK formulation.

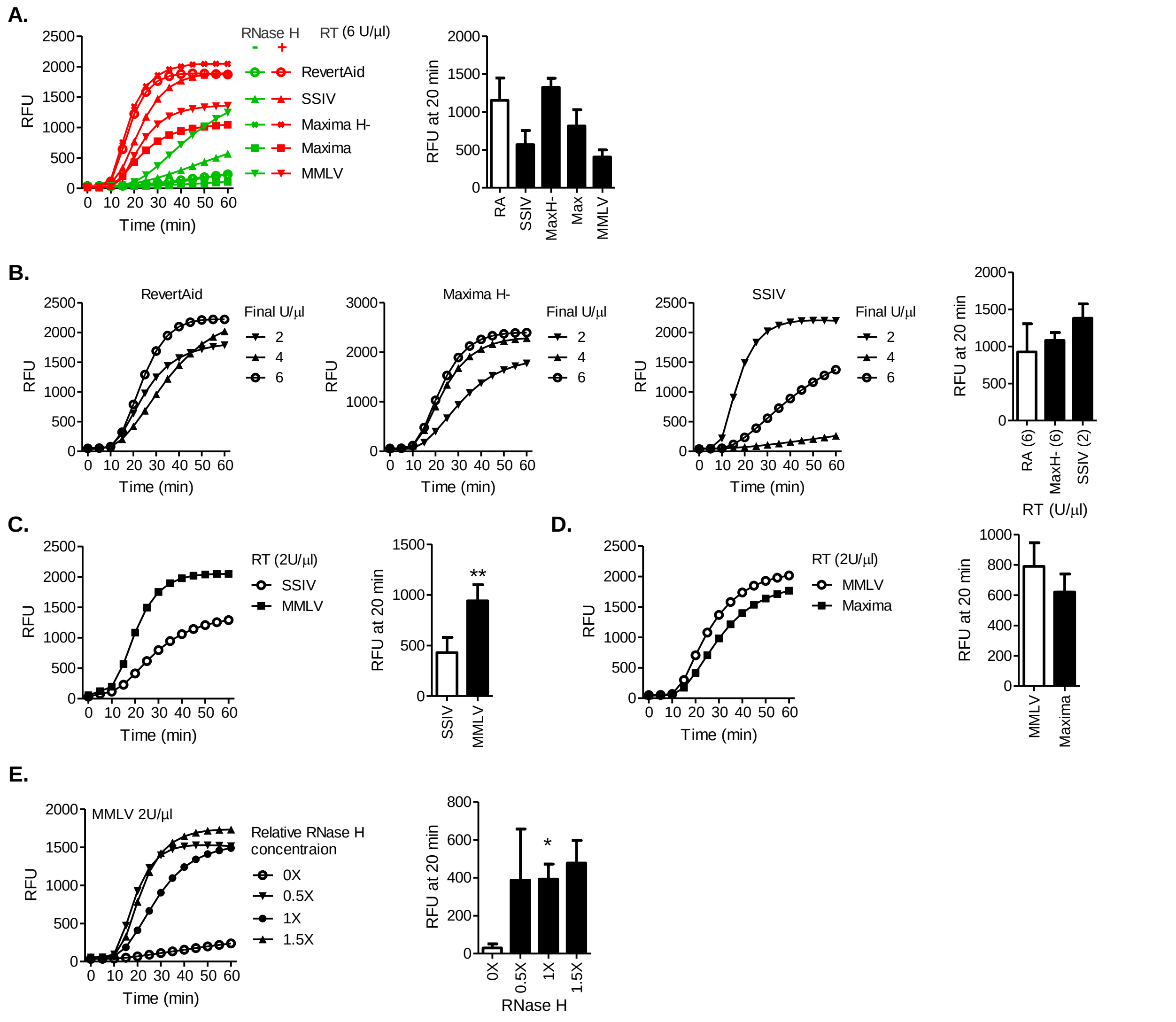

Figure S5. Optimization of reverse transcription (RT) to facilitate RPA. A. RNase H enhances one-pot SHERLOCK. SHERLOCK reactions were performed in the absence (-) or presence (+) of RNase H, in combination with different types of RT enzymes (at indicated final concentration). SSIV = SuperScript IV, Maxima H- = Maxima H Minus and MMLV = M-MuLV. Left: Amplification plot shows relative fluorescent units (RFU) at indicated time points in the SHERLOCK reaction, and represents three independent experiments showing similar patterns. Right: Bar graph shows mean ± SEM, comparing amplification signals at the time point of 20 min. **B.** Effect of RT enzyme concentrations. SHERLOCK reactions were performed using each of the RT enzyme types at indicated concentrations. Amplification plots represent three independent experiments showing similar patterns. Bar graph, based on mean ± SEM, compares the best-performing concentrations for each of enzymes. SSIV at 2 U/µl final concentration was the selected winner among these tested conditions. **C and D.** MMLV is an optimal RT enzyme. Further comparisons of RT enzyme conditions were performed and analyzed as in B. Graphs were based on five (in C) and six (in D) independent experiments, respectively. ** $p < 0.01$, significant difference was found by paired *t* test. **E.** Effect of RNase H concentrations. SHERLOCK reactions were performed under each of the indicated relative concentrations of RNase H. 1× = standard concentration (1.6 µl of 5 U/µl RNase H stock in a 80 µl SHERLOCK reactions. Graphs were based on four independent experiments. * $p < 0.05$, significant difference was found by paired *t* test between the conditions of 0× and 1×.

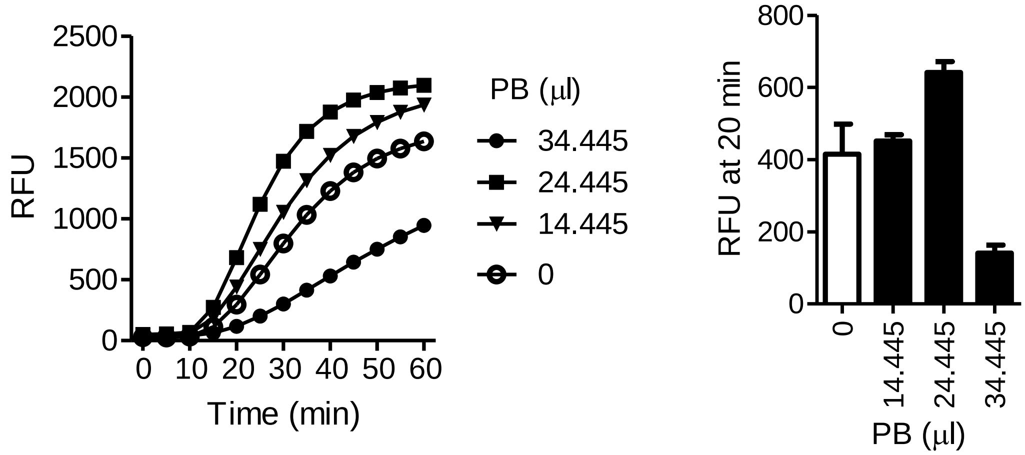

Figure S6. Effect of the protease buffer on one-pot SHERLOCK. The protease buffer (PB) is a candidate extraction-free sample buffer (detailed in Materials and Methods S1). SHERLOCK reactions (80 μl) including PB at each indicated volume were tested for SARS2 detection. Left: Amplification plot shows relative fluorescent units (RFU) at indicated time points in the SHERLOCK reaction, and represents three independent experiments showing similar patterns. Right: Bar graph (mean± SEM) comparing the amplification signals between the condition with a positive volume of PB added and the condition without PB added (0 μl), at the time point of 20 min. The condition with 24.445 μl PB in a 80 μl total reaction volume was optimal and included in the updated SHERLOCK formulation.

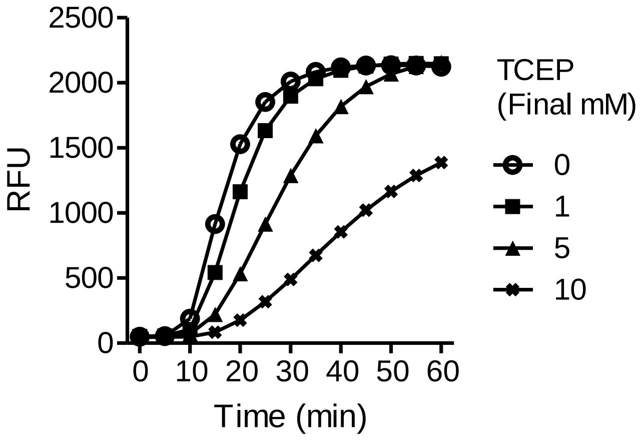

Figure S7. Effect of TCEP on one-pot SHERLOCK. SHERLOCK reactions containing TCEP at each indicated final concentration were tested for SARS2 detection. Amplification plot shows relative fluorescent units (RFU) at indicated time points in the SHERLOCK reaction.

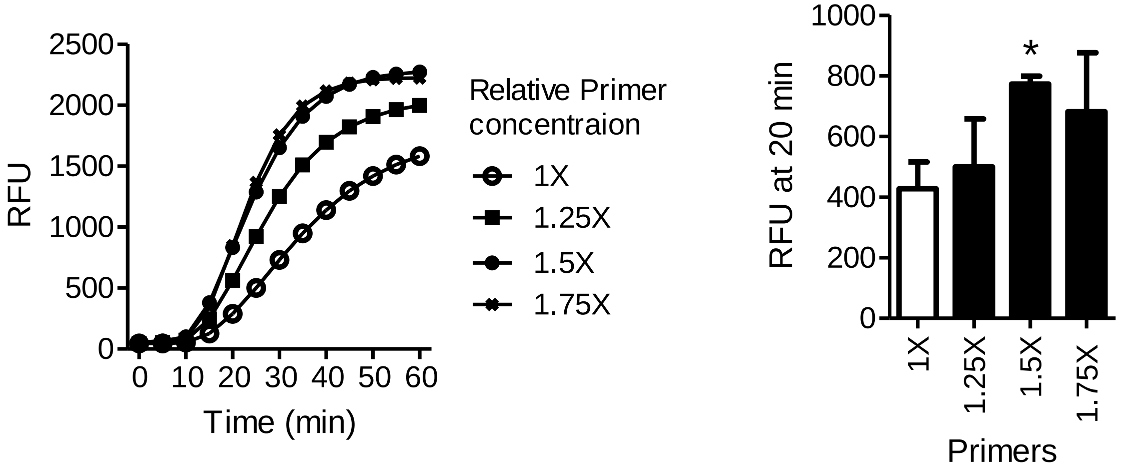

Figure S8. Effect of the RPA primer concentrations on one-pot SHERLOCK. SHERLOCK reactions containing RPA primers at each indicated relative concentration were tested for SARS2 detection. Left: Amplification plot shows relative fluorescent units (RFU) at indicated time points in the SHERLOCK reaction, and represents five independent experiments showing similar patterns. Right: Data were statistically analyzed, comparing the amplification signals between each of the other conditions with the condition of 1× relative concentration, at the time point of 20 min. Bar graph indicates mean ± SEM. Significant difference in RFUs was determined by paired *t* test: * $p < 0.05$. The 1.5× condition demonstrated the best performance and was included in the optimized SHERLOCK formulation. According to this condition, 1.8 µl RPA primer master mix (10 µM T7 promoter-tagged forward primer, 10 µM non-T7 forward primer and 20 µM reverse primer) was used in a 80 µl SHERLOCK formulation.

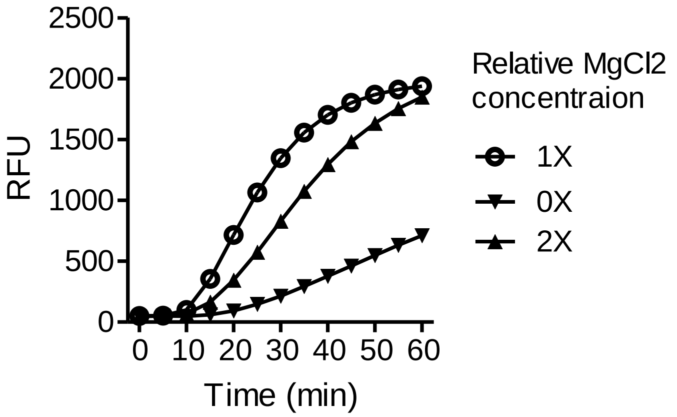

Figure S9. Effect of MgCl2 concentration on one-pot SHERLOCK. SHERLOCK reactions containing MgCl2 at each indicated relative concentration were tested for SARS2 detection. Amplification plot shows relative fluorescent units (RFU) at indicated time points in the SHERLOCK reaction. The 1× condition was kept in the optimized SHERLOCK formulation. According to this condition, 2.25 μl of 200 mM MgCl2 stock solution was used in a 80 μl SHERLOCK formulation.

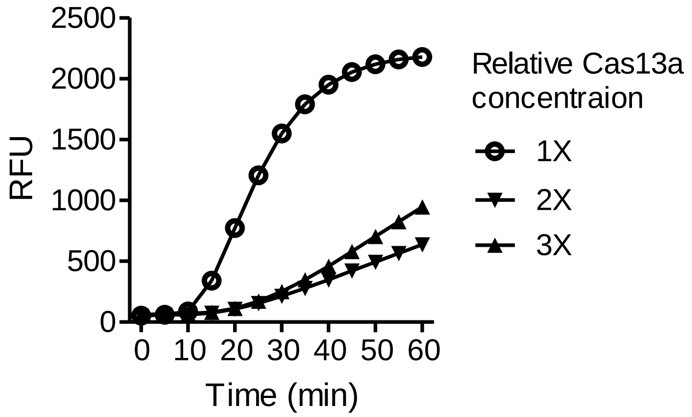

Figure S10. Effect of Cas13a concentration on one-pot SHERLOCK. SHERLOCK reactions containing Cas13a at each indicated relative concentration were tested for SARS2 detection. Amplification plot shows relative fluorescent units (RFU) at indicated time points in the SHERLOCK reaction. The 1× condition was kept in the optimized SHERLOCK formulation. According to this condition, 1.25 µl of LwaCas13a protein (126.6 ng/µl in LwaCas13a storage buffer) master solution was used in a 80 µl SHERLOCK formulation.

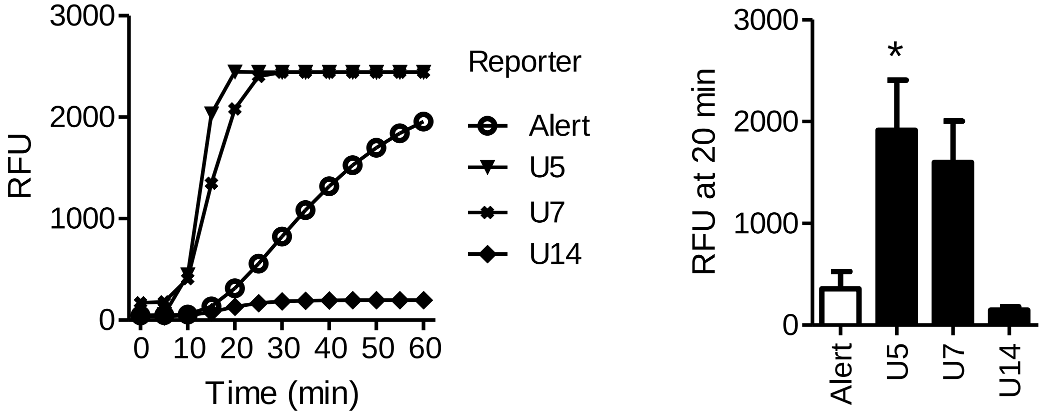

Figure S11. Effect of the fluorescent CRISPR reporter type on one-pot SHERLOCK. SHERLOCK reactions containing each of the indicated reporter types were tested for SARS2 detection. Sequences and labelling dyes of these RNA reporters are listed in Table S1. Alert = RNase Alert v2. Left: Amplification plot shows relative fluorescent units (RFU) at indicated time points in the SHERLOCK reaction, and represents three independent experiments showing similar patterns. Right: Data were statistically analyzed, comparing the amplification signals between each of the other reporters with RNase Alert v2 (the prototype reporter initially used), at the time point of 20 min. Bar graph indicates mean ± SEM. Significant difference in RFUs was determined by paired *t* test: * $p < 0.05$. The U5 reporter demonstrated the best performance and was included in the optimized SHERLOCK formulation.

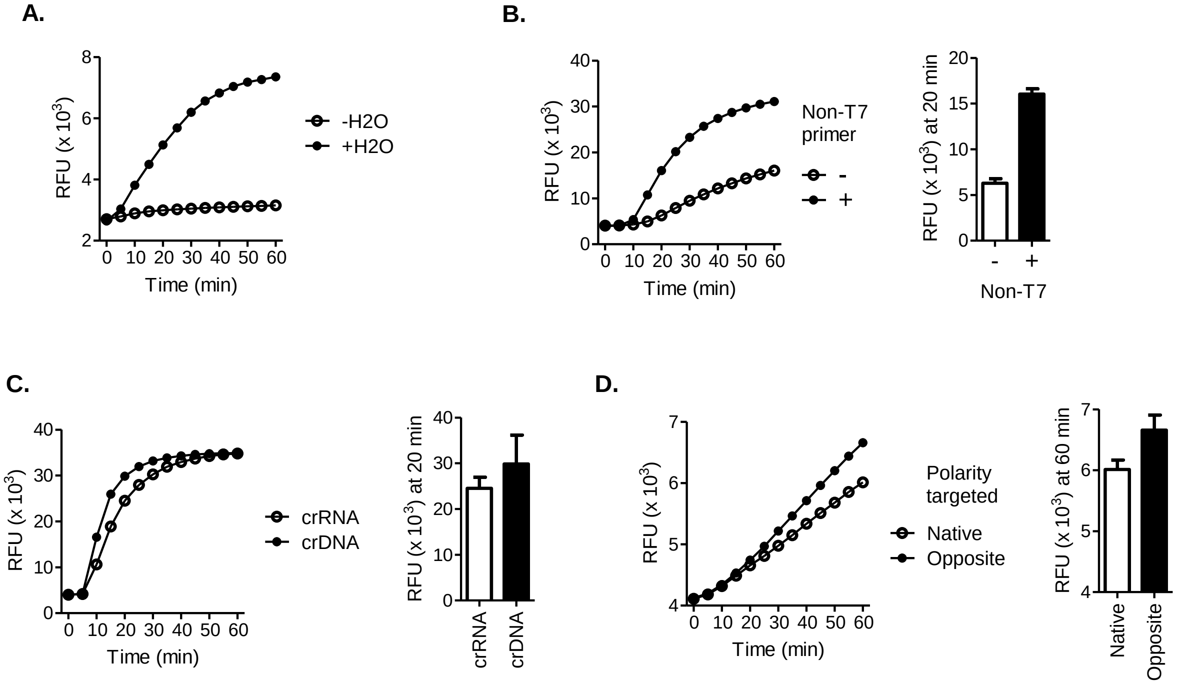

Figure S12. Optimizations are not sequence-specific. Confirmatory tests of major optimization strategies that address interactions between different enzymes/reactions were conducted using additional SHERLOCK sets, which target the E or S gene of SARS-CoV-2 (Table S1). The effect of CRISPR targeting the opposite polarity of SARS2 RNA versus targeting the native polarity on specificity was tested in SHERLOCK sets opE1 and E1 as presented in Figure S2. **A.** Addition of water. SHERLOCK reactions were tested for SARS2 detection in SHERLOCK set opE2, using the base one-pot SHERLOCK formulation without (-H2O) or with (+H2O) the addition of water (30 µl water on top of a 50 µl base reaction). Amplification plot shows relative fluorescent units (RFU) at indicated time points in the SHERLOCK reaction. **B.** Introduction of a non-T7 forward primer. SHERLOCK reactions were performed using SHERLOCK set opS1 in the absence (-) or presence (+) of non-T7 forward primer. Left: Amplification plot shows relative fluorescent units (RFU) at indicated time points in the SHERLOCK reaction. Right: Bar graph indicates mean ± SEM of replicates for the time point of 20 min. **C.** Function of crDNA. SHERLOCK reactions were performed with SHERLOCK set opE2 using either a crRNA or crDNA. Left: Amplification plot shows relative fluorescent units (RFU) at indicated time points in the SHERLOCK reaction. Right: Bar graph indicates mean ± SEM of replicates for the time point of 20 min. **D.** Polarity targeting (effect on signal magnitude). SHERLOCK sets S2 and opS2 were tested in comparison, with CRISPR targeting the native and opposite polarity of SARS2 RNA, respectively. Left: Amplification plot shows relative fluorescent units (RFU) at indicated time points in the SHERLOCK reaction. Right: Bar graph indicates mean ± SEM of replicates for the time point of 60 min.

References

(1) Hansen, S.; Abd El Wahed, A. *Trop Med Infect Dis* **2020**, *5*, 151-165.
(2) Thomas, E.; Delabat, S.; Carattini, Y. L.; Andrews, D. M. *Viruses* **2021**, *13*, 2492.
(3) Rai, P.; Kumar, B. K.; Deekshit, V. K.; Karunasagar, I. *Appl Microbiol Biotechnol* **2021**, *105*, 441-455.
(4) Khan, P.; Aufdembrink, L. M.; Engelhart, A. E. *ACS Synth Biol* **2020**, *9*, 2861-2880.
(5) Duggan, J. *i News* **2022**, https://inews.co.uk/news/pcr-covid-testing-labs-overwhelmed-omicron-surge-1377911.
(6) Gootenberg, J. S.; Abudayyeh, O. O.; Lee, J. W.; Essletzbichler, P.; Dy, A. J.; Joung, J.; Verdine, V.; Donghia, N.; Daringer, N. M.; Freije, C. A.; Myhrvold, C.; Bhattacharyya, R. P.; Livny, J.; Regev, A.; Koonin, E. V.; Hung, D. T.; Sabeti, P. C.; Collins, J. J.; Zhang, F. *Science* **2017**, *356*, 438-442.
(7) Kellner, M. J.; Koob, J. G.; Gootenberg, J. S.; Abudayyeh, O. O.; Zhang, F. *Nat Protoc* **2019**, *14*, 2986-3012.
(8) Myhrvold, C.; Freije, C. A.; Gootenberg, J. S.; Abudayyeh, O. O.; Metsky, H. C.; Durbin, A. F.; Kellner, M. J.; Tan, A. L.; Paul, L. M.; Parham, L. A.; Garcia, K. F.; Barnes, K. G.; Chak, B.; Mondini, A.; Nogueira, M. L.; Isern, S.; Michael, S. F.; Lorenzana, I.; Yozwiak, N. L.; MacInnis, B. L., et al. *Science* **2018**, *360*, 444-448.
(9) Zhang, F.; Abudayyeh, O. O.; Gootenberg, J. S. *Broad Institute* **2020**, https://www.broadinstitute.org/files/publications/special/COVID-19%20detection%20(updated).pdf.
(10) Patchsung, M.; Jantarug, K.; Pattama, A.; Aphicho, K.; Suraritdechachai, S.; Meesawat, P.; Sappakhaw, K.; Leelahakorn, N.; Ruenkam, T.; Wongsatit, T.; Athipanyasilp, N.; Eiamthong, B.; Lakkanasirorat, B.; Phoodokmai, T.; Niljianskul, N.; Pakotiprapha, D.; Chanarat, S.; Homchan, A.; Tinikul, R.; Kamutira, P., et al. *Nat Biomed Eng* **2020**, *4*, 1140-1149.
(11) Li, H.; Bello, A.; Smith, G.; Kielich, D. M. S.; Strong, J. E.; Pickering, B. S. *PLoS Negl Trop Dis* **2022**, *16*, e0010285.
(12) Arizti-Sanz, J.; Bradley, A.; Zhang, Y. B.; Boehm, C. K.; Freije, C. A.; Grunberg, M. E.; Kosoko-Thoroddsen, T. F.; Welch, N. L.; Pillai, P. P.; Mantena, S.; Kim, G.; Uwanibe, J. N.; John, O. G.; Eromon, P. E.; Kocher, G.; Gross, R.; Lee, J. S.; Hensley, L. E.; MacInnis, B. L.; Johnson, J., et al. *Nat Biomed Eng* **2022**, *6*, 932-943.
(13) Daher, R. K.; Stewart, G.; Boissinot, M.; Boudreau, D. K.; Bergeron, M. G. *Mol Cell Probes* **2015**, *29*, 116-121.
(14) Higgins, M.; Stringer, O. W.; Ward, D.; Andrews, J. M.; Forrest, M. S.; Campino, S.; Clark, T. G. *J Mol Diagn* **2022**, *24*, 1207-1216.
(15) Fozouni, P.; Son, S.; Diaz de Leon Derby, M.; Knott, G. J.; Gray, C. N.; D'Ambrosio, M. V.; Zhao, C.; Switz, N. A.; Kumar, G. R.; Stephens, S. I.; Boehm, D.; Tsou, C. L.; Shu, J.; Bhuiya, A.; Armstrong, M.; Harris, A. R.; Chen, P. Y.; Osterloh, J. M.; Meyer-Franke, A.; Joehnk, B., et al. *Cell* **2021**, *184*, 323-333.
(16) Yu, H.; Goodman, M. F. *J Biol Chem* **1992**, *267*, 10888-10896.
(17) Pulsinelli, G. A.; Temin, H. M. *Proc Natl Acad Sci U S A* **1994**, *91*, 9490-9494.
(18) Kharytonchyk, S.; King, S. R.; Ndongmo, C. B.; Stilger, K. L.; An, W.; Telesnitsky, A. *J Mol Biol* **2016**, *428*, 2275-2288.
(19) Stadhouders, R.; Pas, S. D.; Anber, J.; Voermans, J.; Mes, T. H.; Schutten, M. *J Mol Diagn* **2010**, *12*, 109-117.
(20) Lobato, I. M.; O'Sullivan, C. K. *Trends Analyt Chem* **2018**, *98*, 19-35.
(21) *TwistDx* **2023**, https://www.twistdx.co.uk/rpa/using-pcr-primers/.
(22) Piepenburg, O.; Williams, C. H.; Stemple, D. L.; Armes, N. A. *PLoS Biol* **2006**, *4*, e204.
(23) Schultz, S. J.; Champoux, J. J. *Virus Res* **2008**, *134*, 86-103.
(24) Qian, J.; Boswell, S. A.; Chidley, C.; Lu, Z. X.; Pettit, M. E.; Gaudio, B. L.; Fajnzylber, J. M.; Ingram, R. T.; Ward, R. H.; Li, J. Z.; Springer, M. *Nat Commun* **2020**, *11*, 5920.

(25) *ThermoFisher Scientific* **2023**, https://www.thermofisher.com/ca/en/home/life-science/cloning/cloning-learning-center/invitrogen-school-of-molecular-biology/rt-education/reverse-transcriptase-attributes.html.
